# Marine Metabolomics: a Method for Nontargeted Measurement of Metabolites in Seawater by Gas Chromatography–Mass Spectrometry

**DOI:** 10.1128/mSystems.00638-19

**Published:** 2019-12-10

**Authors:** Emilia M. Sogin, Erik Puskás, Nicole Dubilier, Manuel Liebeke

**Affiliations:** a Max Planck Institute for Marine Microbiology, Bremen, Germany; Woods Hole Oceanographic Institution

**Keywords:** marine, metabolomics, microbial ecology, exometabolomics

## Abstract

Microbial communities exchange molecules with their environment, which plays a major role in regulating global biogeochemical cycles and climate. While extracellular metabolites are commonly measured in terrestrial and limnic ecosystems, the presence of salt in marine habitats limits the nontargeted analyses of the ocean exometabolome using mass spectrometry (MS). Current methods require salt removal prior to sample measurements, which can alter the molecular composition of the metabolome and limit the types of compounds detected by MS. To overcome these limitations, we developed a gas chromatography MS (GC-MS) method that avoids sample altering during salt removal and that detects metabolites down to nanomolar concentrations from less than 1 ml of seawater. We applied our method (SeaMet) to explore marine metabolomes *in vitro* and *in vivo*. First, we measured the production and consumption of metabolites during the culture of a heterotrophic bacterium, Marinobacter adhaerens. Our approach revealed successional uptake of amino acids, while sugars were not consumed. These results show that exocellular metabolomics provides insights into nutrient uptake and energy conservation in marine microorganisms. We also applied SeaMet to explore the *in situ* metabolome of coral reef and mangrove sediment porewaters. Despite the fact that these ecosystems occur in nutrient-poor waters, we uncovered high concentrations of sugars and fatty acids, compounds predicted to play a key role for the abundant and diverse microbial communities in coral reef and mangrove sediments. Our data demonstrate that SeaMet advances marine metabolomics by enabling a nontargeted and quantitative analysis of marine metabolites, thus providing new insights into nutrient cycles in the oceans.

**IMPORTANCE** Nontargeted approaches using metabolomics to analyze metabolites that occur in the oceans is less developed than those for terrestrial and limnic ecosystems. One of the challenges in marine metabolomics is that salt limits metabolite analysis in seawater to methods requiring salt removal. Building on previous sample preparation methods for metabolomics, we developed SeaMet, which overcomes the limitations of salt on metabolite detection. Considering that the oceans contain the largest dissolved organic matter pool on Earth, describing the marine metabolome using nontargeted approaches is critical for understanding the drivers behind element cycles, biotic interactions, ecosystem function, and atmospheric CO_2_ storage. Our method complements both targeted marine metabolomic investigations as well as other “omics” (e.g., genomics, transcriptomics, and proteomics) approaches by providing an avenue for studying the chemical interaction between marine microbes and their habitats.

## Abstract (precis)

Nontargeted approaches using metabolomics to analyze metabolites that occur in the oceans is less developed than those for terrestrial and limnic ecosystems. One of the challenges in marine metabolomics is that salt limits metabolite analysis in seawater to methods requiring salt removal. Building on previous sample preparation methods for metabolomics, we developed SeaMet, which overcomes the limitations of salt on metabolite detection. Considering that the oceans contain the largest dissolved organic matter pool on Earth, describing the marine metabolome using nontargeted approaches is critical for understanding the drivers behind element cycles, biotic interactions, ecosystem function, and atmospheric CO_2_ storage. Our method complements both targeted marine metabolomic investigations as well as other “omics” (e.g., genomics, transcriptomics, and proteomics) approaches by providing an avenue for studying the chemical interaction between marine microbes and their habitats.

## INTRODUCTION

Marine microorganisms produce and stabilize the largest pool of organic carbon on Earth by exchanging molecules with their environment (1, 2). Marine microbes are also the basis for maintaining the long-term storage of carbon dioxide (CO_2_) in the oceans, which plays a complex role in biogeochemical cycles with uncertain implications for global climate (3). While metagenomic and metatranscriptomic studies of the ocean, driven by low sequencing costs and projects like Tara Oceans (4) have deepened our knowledge of the identity and activity of marine microbes, these studies are limited in their ability to determine the molecules that contribute to the chemical complexity of marine habitats. New approaches are needed to permit equivalent surveys of the extracellular metabolome or the exometabolome of the ocean.

Exometabolomics provides an opportunity to directly characterize the molecular interaction between microbes and their environment by profiling the types of molecules cellular organisms secrete (5). For instance, in terrestrial and limnic systems, researchers used exometabolomics to study soil organic matter cycling (6, 7), overflow metabolism of cultivable microorganisms (8, 9), and chemical ecology of the environment (10, 11). While intracellular metabolomic analyses of tissues from marine microbial cells to invertebrates are becoming increasingly more common (12–14), the defining characteristic of marine habitats, i.e., high salt concentration, limits exometabolomic analyses of the oceans to studies that require salt removal prior to metabolite extraction (10, 15, 16).

Our knowledge of the metabolite composition of ocean habitats is restricted to methods that require sample preparation techniques that alter their molecular composition or targeted approaches that measure a defined group of metabolites (17–20). The most common environmental profiling strategies in marine ecosystems rely on solid-phase extraction (SPE) techniques to remove salt prior to mass spectrometry (MS) analyses (21, 22). These studies demonstrated the role of microbial communites in transforming readily degradable or labile dissolved organic matter (DOM) into structually complex or refractory compounds that contribute to long-term carbon storage (23). However, the removal of salt from marine samples using SPE is accompanied by the coremoval of small polar compounds, which are the primary components of labile DOM (17, 20). Consequently, SPE-based studies can only detect between 43 and 62% of the dissolved organic carbon pool that contributes to DOM (17, 20), and they do not detect the majority of compounds involved in the central metabolism of cells. Furthermore, current DOM analytical approaches remain inaccessible for many research institutions and projects. This is largely due to high instrumentation costs with relatively low throughput in data acquisition for ultrahigh-resolution MS (e.g., Fourier-transform ion cyclotron MS) and to large sample volume requirements for liquid chromatography-based approaches.

Gas chromatography–MS (GC-MS) analysis, on the other hand, is a high-throughput and widely available analytical method that allows for the detection of primary metabolites, small molecules that occur in central metabolic pathways across biological systems (24, 25). One advantage that GC-MS has over other MS techniques is that it uses electron impact (EI) ionization, which causes fragmentation of molecules. EI sources are better for ionizing metabolites like sugars and sugar alcohols than are electron spray ionization methods. Moreover, they provide consistent fragmentation results that enable spectral matching of molecular libraries to facilitate metabolite identification. These attributes make GC-MS the “workhorse” of analytical chemistry facilities. As such, GC-MS has allowed the identification of metabolites associated with human disease (26), detection of compounds that serve as environmental cues in foraging (27), and description of metabolic fluxes within and between cells (28), and it is used for environmental profiling of soils and microbial activity on land (6, 29). Despite its power to detect metabolites involved in central metabolism, exometabolomic studies using GC-MS from marine habitats are absent due to the inhibitory effects of salt on direct sample analysis.

The ocean metabolome remains largely undefined, despite a growing field of research exploring the molecular composition of DOM (1, 2, 21, 22). To expand our interpretations of ocean metabolism, new cost-effective, high-throughput, and nontargeted workflows are needed to complement current DOM analytical approaches. Here, we present SeaMet, a marine metabolomics method that builds on previous GC-MS sample derivatization methods to enable metabolite detection in seawater. SeaMet avoids sample preparation approaches that coremove metabolites with salt, such as SPE. As such, it complements current exometabolomic methods for marine samples by capturing compounds that are commonly missed during SPE sample preparation. Using SeaMet, we demonstrate how our method can enhance our understanding of microbial metabolism in culture experiments and profiling of marine habitats.

## RESULTS AND DISCUSSION

**FIG 1:**
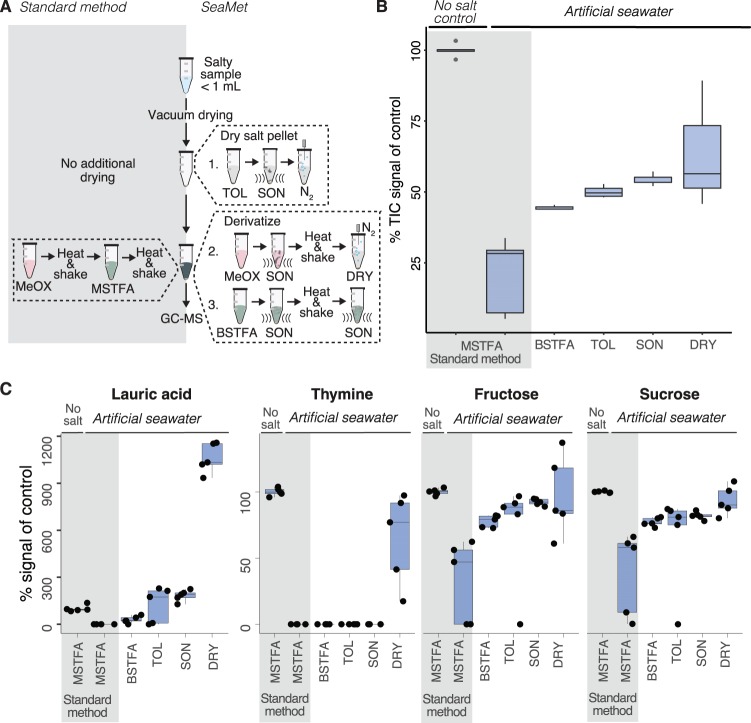
How SeaMet works. (A) Modifications to the standard two-step methoximation (MeOX)- trimethylsilylation (TMS) derivatization protocol include key steps that enhance metabolite signal detection in seawater as shown in panel B. Steps modified from the standard method include a switch in derivatization reagents from MSTFA to BSTFA, removal of water azeotropically by the addition of toluene (TOL) to the salt pellet and subsequent removal under N_2_ gas, ultrasonication (SON) after the addition of TOL, MeOX, BSTFA, and after BSTFA derivatization, and drying (DRY) of the pyridine after the MeOX derivatization prior to BSTFA addition. (B) Box plots showing changes in total ion chromatogram (TIC) signals of a metabolite mixture after GC-MS data acquisition. The results are from a mixture of 45 metabolites representing a broad scope of metabolite classes (Table S1) dissolved in 0.5 ml of seawater (*n *= 5) relative to the average of the no-salt control. (C) Box plots showing increases in peak areas of individual metabolites relative to the no-salt control for lauric acid, thymine, fructose, and sucrose.

SeaMet modifies the well-established two-step derivatization procedure, which permits the detection of nonvolatile primary metabolites using GC-MS and involves methoximation followed by trimethylsilylation (30). Like other GC-MS sample preparation techniques (31, 32), SeaMet removes liquid through vacuum drying prior to derivatization, a process that results in a salt pellet when working with marine samples, which restricts MS analysis. Our preliminary tests suggested that water locked within the dried salt crystals hindered the chemical reactions needed for GC-MS (see Fig. S1 in the supplemental material). Our method overcomes this limitation by first eliminating residual water within the salt crystals and then extracting metabolites into the derivatization reagents (Fig. 1A).

10.1128/mSystems.00638-19.1 FIG S1 Salt and water inhibit metabolite derivatization reactions. Total detected ion signals are negatively related to increasing concentrations of salt (A) and water (B) for both MSTFA (red circles) and BSTFA (blue circles) derivatization reagents. Signals intensities from the metabolite mixture (Table S1) are relative to control samples (A, no salt; B, no water). To avoid damage to the GC-MS instrumentation, only 1 replicate/condition was analyzed. Download FIG S1, EPS file, 0.2 MB. Copyright © 2019 Sogin et al. 2019 Sogin et al. This content is distributed under the terms of the Creative Commons Attribution 4.0 International license.

10.1128/mSystems.00638-19.6 TABLE S1 Metabolite database of 113 metabolites detected in artificial seawater using SeaMet. Compounds used for testing and method development steps are indicated with their retention times. Metabolites with multiple retention times represent different TMS derivatives. Kyoto Encyclopedia of Genes and Genomes (KEGG) identifiers and the associated BRITE functional hierarchy or metabolite class of each compound are indicated. Download Table S1, PDF file, 0.1 MB. Copyright © 2019 Sogin et al. 2019 Sogin et al. This content is distributed under the terms of the Creative Commons Attribution 4.0 International license.

We used a mixture of 45 different metabolites (Table S1) dissolved in artificial seawater, so each compound had a final concentration of 0.4 mM (analogous to a high-DOM sample representative of a cell culture) to document the increased performance in metabolite detection of our method at each development step. We chose to document increases in metabolite signals using high concentrations of compounds dissolved in artificial seawater in order to first observe signal improvements and to control the background conditions of our samples (e.g., sample salinity and molecular diversity of the matrix). Overall, SeaMet increased the total signal intensity on average by 42% and up to 89% for high-salinity samples in comparison to the standard GC-MS sample preparation (Fig. 1B). SeaMet not only increased the total ions detected, but it also increased the signals from individual metabolites in our samples for each protocol modification step (Fig. 1C).

We first replaced the most commonly used trimethylsilylation reagent, *N*-methyl-*N*-(trimethylsilyl)trifluoroacetamide (MSTFA) (32), with one that is less susceptible to inhibition by water, *N*,*O*-Bis(trimethylsilyl)trifluoroacetamide (BSTFA), which resulted in higher metabolite signals (Fig. S1B). To eliminate water from the samples, we increased the speed vacuum drying time from 4 to 8 h and integrated a toluene drying step that is used in urine-based metabolomic analyses (31). We further enhanced metabolite signals by treating the salt pellet to a combination of ultrasonication and vortexing after the addition of toluene and both derivatization reagents, and following the completion of the trimethylsilylation reaction. These steps break apart the salt crystals and release water into the toluene to enhance salt drying and metabolite extraction. Finally, following a recently described method for improving GC-MS metabolite detection regardless of sample type (33), we included an additional step between the methoximation and trimethylsilylation derivatization reactions and evaporated the first derivatization reagent under N_2_ gas (see Fig. 1B for total signal improvements of each step for the metabolite mixture).

**FIG 2:**
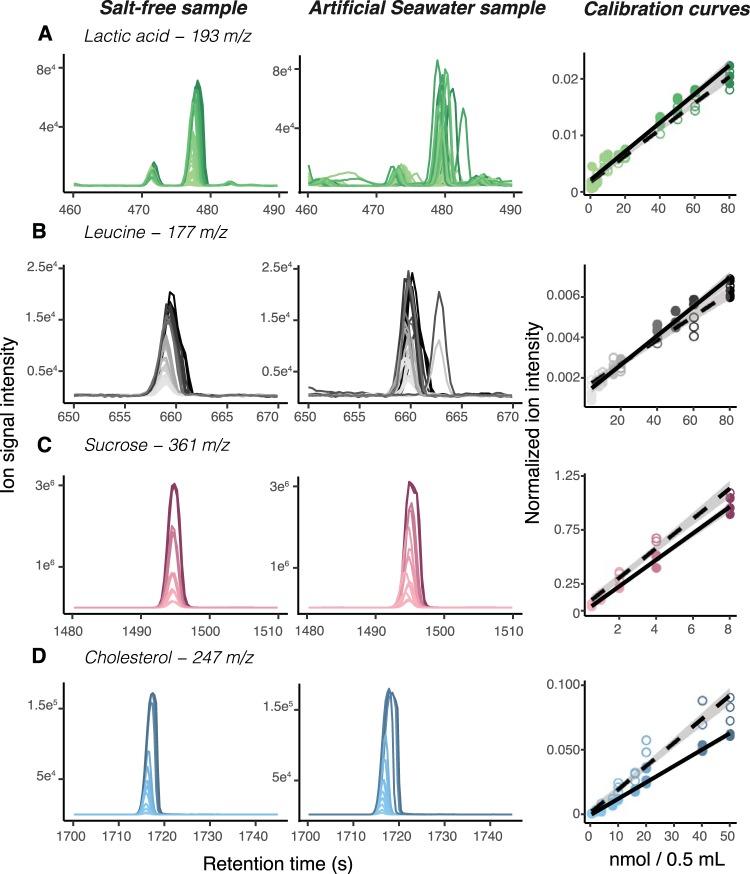
Metabolite detection and quantification in artificial seawater using SeaMet in comparison to salt-free water. (A to D) Extracted ion chromatograms for select metabolites in salt-free and artificial seawater demonstrate reproducible metabolite detection across concentration gradients, as shown in associated calibration curves on the right. Spectra and points are shaded in scale with sample concentrations, where more-concentrated samples are represented by darker colors. Calibration curves were collected in a mixture of 45 metabolites (Table S1). Open circles, artificial seawater samples; filled circles, salt-free samples.

Overall, SeaMet allowed us to detect significant increases in metabolite abundances across molecular classes compared to the standard method for GC-MS sample preparation (adjusted *P* < 0.05; mean fold change across all ions, 323; Fig. 2A and S2) (31). This included measurement of organic acids, amino acids, and fatty acids, as well as sugars and their stereoisomers, sugar alcohols, and sterols (Table S1). We also tested our method to ensure that detected metabolite signals are not artifacts of compound degradation related to sample treatment. We did not observe increases in ion intensities related to the presence of complex biopolymers, specifically, a marine polysaccharide (laminarin) and proteins (bovine serum albumin), compared to samples containing only artificial seawater (adjusted *P* > 0.05; Fig. S3A).

10.1128/mSystems.00638-19.2 FIG S2 Calibration curves for individual metabolites in salt-free and artificial seawater (ASW). Calculated calibration curves were compared for compounds that were detected under both salt-free and ASW conditions (*n* = 3 for each concentration). Gray shading represents 90% confidence intervals, and points are fitted using a linear regression. The model results are reported in Table S2. Download FIG S2, PDF file, 0.1 MB. Copyright © 2019 Sogin et al. 2019 Sogin et al. This content is distributed under the terms of the Creative Commons Attribution 4.0 International license.

10.1128/mSystems.00638-19.3 FIG S3 SeaMet does lead to the breakdown of complex proteins or carbohydrates. (A and B) Total ion chromatogram cloud plots of experimental samples (top profiles) containing laminarin and bovine serum albumin prepared with SeaMet (A) and dried down in a speed vacuum at 60°C and 16 h (B) in comparison to control samples containing only artificial seawater (bottom). Detailed insets show little variation in metabolite profiles for both comparisons. Significant differences (adjusted *P* < 0.05) in ion intensities were only observed in panel B (sizes of bubbles are scaled to fold change differences in ion abundances). Peaks matching NIST annotations include 1, tetradecanoic acid; 2, unknown peak; 3 and 4, octadecatrienoic acid; 5, octacosane. Only when we increased the sample drying temperature from 45°C to 60°C and the time from 8 h to 16 h did we observe changes in our metabolite profiles (adjusted *P* < 0.05; FC, >2). Download FIG S3, EPS file, 3.0 MB. Copyright © 2019 Sogin et al. 2019 Sogin et al. This content is distributed under the terms of the Creative Commons Attribution 4.0 International license.

To determine the quantitative capabilities of SeaMet, we used a metabolite mixture (45 metabolites spanning 9 compound classes) and added different amounts of metabolite stock solution (0.4 mM) to reach concentration levels between 1 μM and 16 μM to both artificial seawater (ASW) and natural seawater (NSW). Our detection limits were in the nanomolar range per 0.5 ml of sample and comparable to those of targeted techniques for marine ecosystems that were developed to quantify single compounds from specific molecular classes (Tables S2 and S3). Our method provides reproducible quantification across a select set of metabolites (*r*^2^ > 0.7) and gives a linearity and dynamic range (Table S2) in ASW similar to those of salt-free samples prepared with the standard GC-MS derivatization method (Fig. 2 and S2). Moreover, we demonstrate that SeaMet reduces the variation in ion detection for individual metabolites (Welch’s *t* test, *P* < 0.01 across all ions at 8 μM; average % coefficient of variation of ASW [CV_ASW_], 20.2 ± 0.78; average % CV_no-salt_, 23.5 ± 0.72) compared to that of salt-free samples prepared with the standard GC-MS derivatization procedure (Table S2). We further explored the influence of increasing DOM concentrations on our capacity to detect individual compounds in natural seawater. To this end, we prepared replicate natural seawater samples containing various concentrations of Marine broth (0, 50, and 250 μl in 0.5 ml of NSW) and added our metabolite mixture to each sample. Overall, the variation in the metabolite detection of most compounds (except some amino acids that are relatively more difficult to detect using GC-MS) in natural seawater was low and consistent between samples that had no additional DOM, low DOM, and high DOM (Fig. S4).

10.1128/mSystems.00638-19.4 FIG S4 SeaMet can recover individual metabolites from low- to high-carbon samples. (A and B) Increasing DOM concentrations, as represented by increases in the amount of Marine broth (MB) added to natural seawater (NSW), only impacts the ability to accurately recover valine and leucine (A), which tend to have higher coefficient of variation values (B). Download FIG S4, PDF file, 0.1 MB. Copyright © 2019 Sogin et al. 2019 Sogin et al. This content is distributed under the terms of the Creative Commons Attribution 4.0 International license.

10.1128/mSystems.00638-19.7 TABLE S2 Quantification ions, calibration coefficients, and retention of metabolites from each compound class under no-salt and salt conditions. The minimum and maximum calibration points of select compounds from representatives of each metabolite class in artificial seawater are reported. The lowest concentration at which signals were observed is reported for select compounds. Download Table S2, XLSX file, 0.1 MB. Copyright © 2019 Sogin et al. 2019 Sogin et al. This content is distributed under the terms of the Creative Commons Attribution 4.0 International license.

10.1128/mSystems.00638-19.8 TABLE S3 Targeted techniques for the quantification of specific metabolite classes in seawater. Download Table S3, PDF file, 0.1 MB. Copyright © 2019 Sogin et al. 2019 Sogin et al. This content is distributed under the terms of the Creative Commons Attribution 4.0 International license.

In contrast to previously published techniques, which require at least an order-of-magnitude-higher sample volumes (17, 20), SeaMet only requires 0.5 ml to 1 ml of seawater for metabolite detection. Using SeaMet, we measured 113 metabolite standards in seawater, representing major metabolite groups involved in primary metabolic pathways (Table S1). The analytical characteristics of the 113 metabolites can be used for more sensitive, targeted GC-MS analyses or for help in identifying metabolites in nontargeted applications. Given that sample matrices are known to impact the ability to accurately quantify individual metabolites regardless of MS approaches, it was beyond the scope of this study to produce calibration curves for all detected compounds. Instead, we recommend to make calibration curves for each metabolite of interest in the desired sample matrix to enhance the quantification success of individual compounds occurring in complex samples.

**FIG 3:**
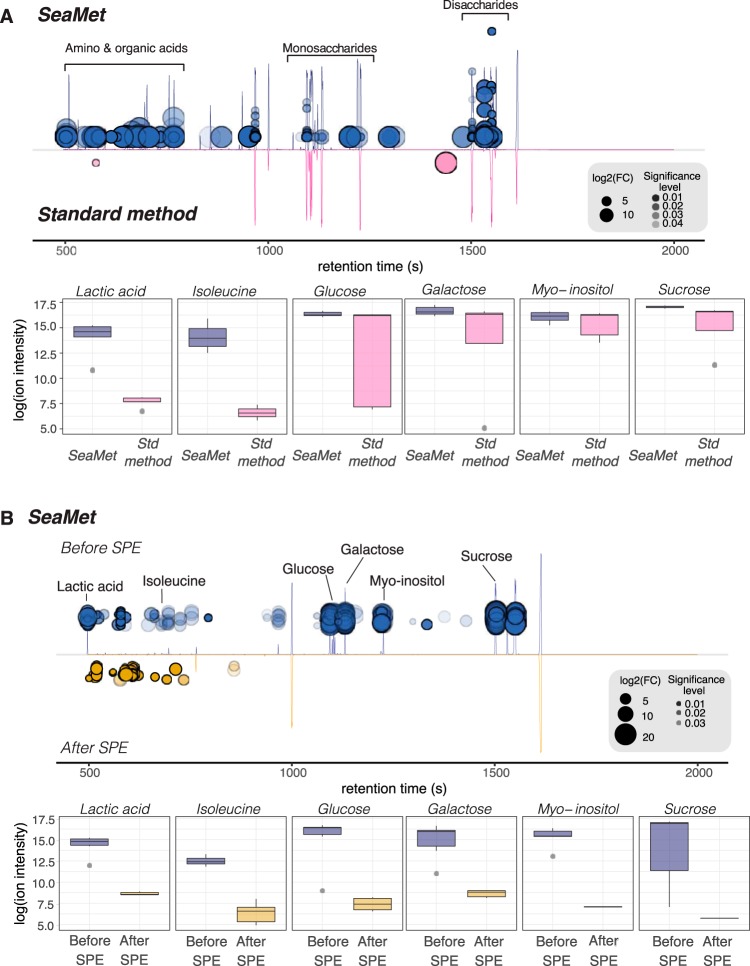
SeaMet enhances the detection of metabolites in marine samples. (A and B) Total ion chromatogram cloud plots from GC-MS profiles of metabolite mixtures indicate significant differences (Benjamini-Hochberg adjusted *P *< 0.05) between ion abundances when comparing SeaMet (blue, top) to the standard (Std) metabolite derivatization (pink, bottom) protocol for GC-MS samples (A) and chromatograms using SeaMet on marine samples before (blue, top) and after (yellow, bottom) solid-phase extraction (SPE) (B). Individual compound box plots are also shown to highlight improvements in metabolite detection using SeaMet. For the cloud plots, larger bubbles indicate higher log_2_(fold changes) between groups, and more intense colors represent lower *t* test *P* values in a comparison of individual feature (*m/z* ions) intensities. Samples prepared with SeaMet had high abundances of organic acids (lactic acid, succinic acid, and fumarate), amino acids (isoleucine, leucine, threonine, and valine), sugar alcohols (myo-inositol and mannitol), and sugars (fructose, glucose, cellobiose, maltose, ribose, galactose, and sucrose) in comparison to SPE-based sample preparation. Representatives of each class are indicated in panel B. To show signal improvement using SeaMet, samples for both comparisons included authentic metabolite standards representing multiple chemical classes.

Given that SeaMet avoids SPE, we assessed how SPE sample treatment affects the ability to detect compounds in marine samples. We compared GC-MS profiles measured with SeaMet before and after salt removal using the most commonly used Bond Elut styrene-divinylbenzene (PPL) SPE columns. Our analyses provide direct evidence that small polar compounds, such as sugars, sugar alcohols, amino acids, and organic acids, were coremoved with salt during SPE sample preparation (Fig. 3B). These results provide further evidence that SeaMet captures compounds commonly missed by SPE-based exometabolomic approaches for marine samples. Furthermore, our analyses complement previous studies indicating that some compound classes are weakly retained during SPE-based sample preparation (20). Consequently, by offering a complementary technique to current DOM approaches, we advance marine metabolomics research by expanding the range of metabolites that can be measured by nontargeted approaches.

**FIG 4:**
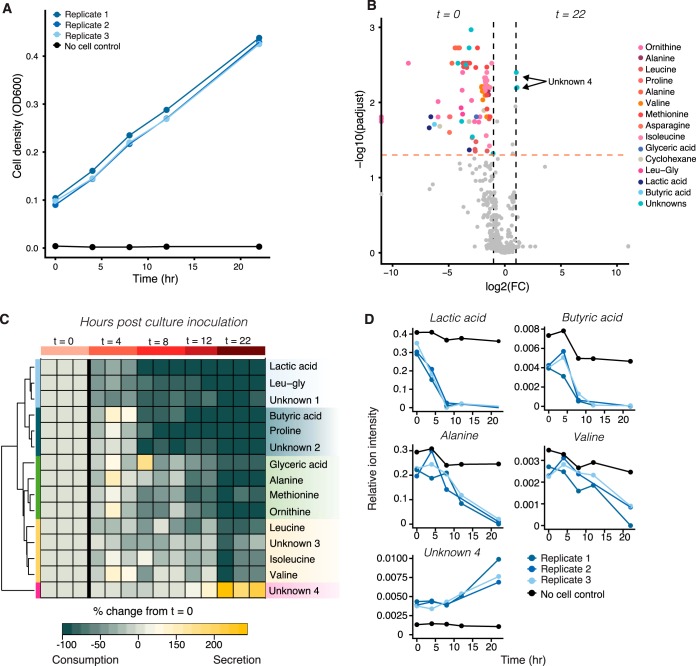
Metabolite consumption and excretion during culture of the marine heterotroph Marinobacter adhaerens. (A) Cell densities increased during the first 22 h of culture growth in Marine broth. (B) Volcano plot showing differences in ion abundances in cell growth medium between the initial and final (22-h) sampling time points. Variables exhibiting high fold change values [log_2_(fold change), >2] and significant differences (adjusted *P* < 0.05) between the two sampling time points are colored according to their metabolite database (NIST) annotation. (C) A heatmap of metabolite abundances after 22 h relative to starting conditions indicates that some compounds, like the dipeptide leucine-glycine (Leu-gly), and lactic acid were taken up before others, such as branched-chain amino acids. After 12 to 22 h of growth, the bacteria excreted an unknown compound predicted to contain an amine group (unknown 4). Hierarchical clustering shows groups of metabolites that changed significantly during growth (colored bars on left; B.H. adjusted *P* < 0.05; fold change, >2). These metabolite groups represent successive stages in *M. adhaerens* consumption and production of marine broth components. (D) Relative ion abundances over time for select metabolites from each cluster group shown in panel C. The blue lines represent biological replicate cultures, while the black lines show results from a control sample with no cell addition. Low variation among biological replicates highlights the reproducibility of SeaMet.

To demonstrate the capability of SeaMet in characterizing the metabolism of marine bacteria, we monitored changes in the extracellular metabolome during the growth of a heterotrophic gammaproteobacterium, Marinobacter adhaerens, that occurs in aggregation with diatoms throughout the North Sea. Using SeaMet, we simultaneously observed 681 peaks (as deconvolved by AMDIS) matching 298 NIST database annotations (Table S4). Using an untargeted approach with all peaks, we detected significant changes in the metabolite composition of marine culture medium during the bacterium’s initial growth phase (adjusted *P* < 0.05; Fig. 4 and S5). The bacterium took up different carbon and nitrogen resources in a cascade-like fashion, and later in growth, it began excretion of an undescribed compound that is predicted to contain an amine group (Fig. 4C and D and S4). By measuring multiple metabolite classes (e.g., sugars, amino acids, organic acids) in a single analytical run, our results revealed that *M. adhaerens* preferentially took up amino acids over readily available sugar compounds (e.g., trehalose; Fig. S5). Previous proteomic results indicated that *M. adhaerens* had a high number of expressed amino acid uptake transporters (34). Our results expand on these findings by (i) highlighting which amino acids *M. adhaerens* prefers, (ii) providing experimental evidence that this heterotroph does not take up sugars during growth in complex medium, despite the genomic ability to use them in their metabolism (35), and (iii), showcasing that *M. adhaerens* participates in the successional uptake of resources. Successional dynamics in substrate use is a common energy conservation mechanism in bacteria (36) and affects central carbon and nitrogen dynamics during growth. *M. adhaerens*, like many other bacteria, participates in the release of organic carbon, which can be metabolized by other microorganisms or will contribute to the complexity of refractory DOM.

10.1128/mSystems.00638-19.5 FIG S5 Extracellular metabolite levels shift with cell culture density. Metabolite relative abundances for each cell culture and no-cell control are plotted through time. Only metabolites that significantly (adjusted *P* < 0.05) varied with time in replicate culture experiments are plotted for clarity. Download FIG S5, PDF file, 0.1 MB. Copyright © 2019 Sogin et al. 2019 Sogin et al. This content is distributed under the terms of the Creative Commons Attribution 4.0 International license.

**FIG 5:**
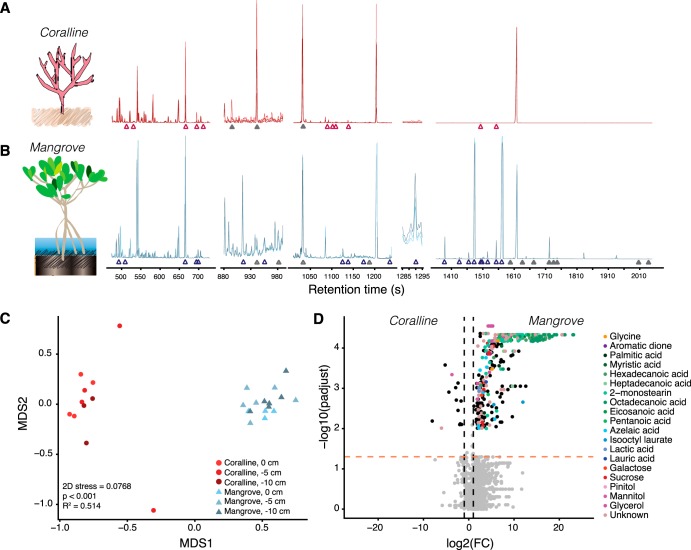
Metabolite profiles from marine habitats acquired with SeaMet. (A and B) GC-MS metabolomic profiles from coralline (A) and mangrove (B) sediment porewaters showed high concentrations of identified metabolites (open triangles), e.g., fatty acids and sugars that explain multivariate differences in compositions shown in panel C. Profiles also revealed unknown peaks (filled triangles) for which no matches were found in public databases (Table S4). (C) Bray-Curtis-informed nonmetric multidimensional scaling analysis of sediment porewater metabolomic profiles from coralline (red) and mangrove (blue) habitats across sediment depths. ADONIS *P* and *R*^2^ values showed a significant correlation between sampling location and metabolite composition. (D) Volcano plot showing differences in detected ion abundances between habitats. Significant ions (adjusted *P* < 0.05) with a log_2_(fold change) of >2 are shaded according to their metabolite database (NIST) annotation based on the combined mass spectra of the deconvolved peak. All ions detected for each peak are represented in the volcano plot.

10.1128/mSystems.00638-19.9 TABLE S4 AMDIS annotations from cell culture and environmental porewater data. For unknowns, compounds driving separation in metabolite space, the Kovats retention time index, Golm predicted functional groups, and BinBase splash identifiers are reported. Compounds that did not match NIST database entries are labeled as “unknown,” followed by their retention time. *, compounds that drove variation between sampling habitats, as detected in Fig. 5D. Metabolites driving separation between sampling data sets that were not identified by AMDIS were further identified through comparison to NIST in Mass Hunter. Download Table S4, PDF file, 0.3 MB. Copyright © 2019 Sogin et al. 2019 Sogin et al. This content is distributed under the terms of the Creative Commons Attribution 4.0 International license.

Considering that SeaMet covers a range of molecular classes underlying primary metabolism, we were able to capture changes in marine chemistry as a result of physiology. Therefore, we predict that our method will enable the measurement of changes in the labile DOM pool as it becomes more refractory. Given the ease in applying our method to culture studies, it is possible to integrate SeaMet with other MS (e.g., proteomics and other DOM-based metabolomics) and sequencing-based approaches to illuminate microbial physiology in the marine environment. By identifying and quantifying metabolites that are consumed and excreted in cultivable marine bacteria, our method expands our understanding of key primary compounds involved in the transformation of organic matter in the ocean.

To test the ability of our workflow to assess complex environmental metabolomes, we applied SeaMet to porewater samples from coralline and mangrove sediments. Coral reefs and mangroves, two globally important coastal ecosystems, contain many biological compounds that remain undescribed. It is essential to characterize the metabolome of these habitats to understand the role of these ecosystems in biogeochemical cycling.

Our approach detected 167 and 211 deconvolved metabolite peaks from coralline and mangrove porewater profiles (Fig. 5A and B), including sugars, amino acids, organic acids, fatty acids, and background signals. From these peaks, we were able to identify 77 in coralline sediments and 117 in mangroves using AMDIS (Table S4). In order to take a nontargeted analytical approach, we reprocessed the raw metabolite profiles using XCMS (see supplemental material for our peak picking script). We used a volcano plot to compare individual ion abundances from each detected peak to show that diverse and abundant sugars (e.g., sucrose and trehalose) from sediment porewaters adjacent to corals, as well as fatty acids (e.g., hexadecanoic acid and steric acid) from porewaters next to mangroves, drove the observed differences between habitats (ADONIS, *P* < 0.001, *R*^2^ = 0.514; Fig. 5C and D and Table S4). Furthermore, our data suggest that metabolite profiles are relatively stable within each habitat, as we did not observe significant differences (ADONIS, *P* > 0.05) between sampling spots or depths.

Given that corals and mangroves thrive in oligotrophic waters and their associated sediments harbor diverse, abundant, and metabolically active microorganisms (37, 38), we were surprised to measure high levels of metabolites (e.g., simple sugars like trehalose and galactose and amino acids like glycine and alanine) that are typically consumed in primary metabolism. Metabolomic analyses of marine sediments (in bulk) have also detected high abundances of primary metabolites (39, 40), suggesting that sediment habitats, which are globally home to an estimated 2.9 × 10^29^ microbial cells (41), contain many different types of compounds that drive microbial community metabolism. These data call for a reexamination of carbon sequestration in coastal sediments using techniques that can identify and quantify the accumulation of liable metabolites.

A large portion of ocean chemistry remains unannotated, reflecting one of the central challenges in metabolomics research (42). As an example, our samples from sediment porewaters of mangroves and coral reefs revealed 11 metabolites driving variation between habitats that did not match public database entries (Fig. 5 and Table S4) (43, 44). The paucity of annotated marine metabolites in the databases is mainly due to the technical challenges involved with measuring water samples containing high salt concentrations. The development of new analytical approaches will help efforts to improve metabolite annotation in marine systems. By providing a new method to measure a broad scope of the marine metabolome, we offer an avenue to identify molecules from marine environments and expand existing mass spectrometry databases that aim to characterize chemical space across ecosystems. For instance, we deposited the data presented here, including mass spectra of authentic standards to MetaboLights, to help facilitate the annotation of GC-MS data sets from marine samples (see “Data availability,” below).

SeaMet is a marine metabolomics workflow that enables the analysis of primary metabolism in the oceans. It is time efficient, allows the detection of diverse metabolite classes in a single run, and expands the analytical window for molecules that can be detected within marine samples. This advance enables nontargeted metabolomics for marine ecosystems using a low-cost, easy-to-use GC-MS platform. Moreover, SeaMet is independent of GC-MS instrumentation, allowing it to be combined with time-of-flight or Orbitrap GC-MS setups to provide faster analysis time and higher mass resolving power to improve metabolite identification. We expect that our marine metabolomics workflow will enable the exploratory analysis of metabolites occurring in seawater and thereby advance our understanding of the ocean’s vast and largely unexplored metabolome.

## MATERIALS AND METHODS

### Reagents and experimental sample preparation.

The derivatization chemicals trimethylsilyl-*N*-methyl trifluoroacetamide (MSTFA) and *N,O*-Bis(trimethylsilyl)trifluoroacetamide (BSTFA) were obtained from CS-Chromatographie Service and pyridine obtained from Sigma-Aldrich at >99.98% purity. Methoxyamine hydrochloride (MeOX; Sigma-Aldrich) aliquots were further dried at 60°C in a drying oven for 1 h to remove residual moisture. Artificial seawater (ASW) was prepared within the range of natural salinity (36‰) by dissolving (per liter of water) 26.37 g sodium chloride, 6.8 g magnesium sulfate heptahydrate, 5.67 g magnesium chloride hexahydrate, 1.47 g calcium chloride, 0.6 g potassium chloride, and 0.09 g potassium bromide. Following autoclave sterilization, pH was adjusted to 7.7 using sodium hydroxide. One milliliter of the following supplements and solutions were added: 150 mM monopotassium phosphate, 500 mM ammonium chloride (pH 7.5), trace element solution (7.55 μM iron sulfate, 4.85 μM boric acid, 0.51 μM manganese chloride, 0.8 μM cobalt chloride, 0.1 μM nickel chloride, 0.06 μM copper chloride, 0.5 μM zinc sulfate, 3.72 μM sodium permanganate in Milli-Q [MQ] water), selenite-tungstate solution (10 μM sodium hydroxide, 0.11 μM sodium selenite, 0.02 μM sodium tungstate dihydrate in Milli-Q water), vitamin solution (10 μM 4-aminobenzoic acid, 10 μM d-biotin, 100 μM nicotinic acid, 50 μM calcium d-pantothenate in 10 mM sodium phosphate buffer [pH 7.1]), thiamine solution (250 μg/liter thiamine chloride dihydrochloride in 25 mM sodium phosphate buffer [pH 3.4]), B_12_ solution (50 μg/liter cyanocobalamin in Milli-Q water), and 0.21 g sodium bicarbonate (45). Ultrapure water (MQ) was prepared by purifying deionized water with an Astacus membraPure system (conductivity at 25°C = 18.3 mΩ × cm).

Metabolite standards were obtained from commercial sources (Table S1) and combined into mixtures in which each compound had a final concentration of 0.4 mM. Metabolite mixtures were prepared to (i) test the effect of salt and water on metabolite detection, (ii) develop SeaMet, our marine metabolomics workflow, (iii) compare metabolite detection before and after solid-phase extraction (SPE)-based sample preparation, and (iv) quantify the detection limits of specific compound classes (Table S2). Finally, multiple mixtures were prepared to document the retention times of 113 standards dissolved in ASW using SeaMet (Table S1). Sample aliquots for the above-mentioned experiments were prepared by drying down 200 μl of the mixture in a speed vacuum concentrator (Eppendorf Concentrator plus, 2.5 h, 45°C, vacuum-aqueous [V-AQ]) for all experiments except SPE comparison and quantification of detection limits. Each resulting compound had a final concentration of 80 nmol in 0.5 ml (16 μM). For the SPE comparison experiment, 400 μl of the mixture was dried down and resuspended in 2 ml. For the quantification of metabolite classes, a serial dilution of the mixture was prepared to obtain between 0.5 nmol and 80 nmol each compound, representing the dynamic range of the method. Dried metabolites were resuspended in 0.5 ml of either MQ water, ASW, or NSW in order to obtain concentrations between 1 μM and 16 μM. In addition, to determine the detection limits of the method, 0.031 nmol select compounds was dried down and resuspended on 0.5 ml of ASW. All mixture aliquots used to obtain calibration curves were prepared using SeaMet.

### SeaMet metabolite derivatization.

To prepare marine samples for gas chromatography–mass spectrometry (GC-MS) analysis, 0.5 to 1 ml of a saltwater sample or experimental mixture dissolved in ASW was dried in a speed vacuum concentrator for 8 h (Eppendorf Concentrator plus, 45°C, V-AQ). To further remove residual water locked within the salt pellet, 250 μl of toluene (99.8%, <0.2% water) was added to each sample, and the mixture was ultrasonicated for 10 min at maximum intensity. The toluene was subsequently removed under a gentle flow of N_2_ gas. Metabolite derivatization was performed by adding 80 μl of MeOX dissolved in pyridine (20 mg ml^−1^) to the dried pellet. The mixture was ultrasonicated (EMag Emmi-12HC) for 10 min at maximum intensity, briefly vortexed to dissolve the pellet into solution, and subsequently incubated for 90 min at 37°C using a thermal rotating incubator under constant rotation at 1,350 rpm. The pyridine was removed from the sample at room temperature under a gentle flow of N_2_ gas (approximately 1 h). Following the addition of 100 μl of BSTFA, the mixture was ultrasonicated for 10 min at maximum intensity, vortexed, and incubated for 30 min at 37°C using a thermal rotating incubator under constant rotation at 1,350 rpm. The derivatized mixture was ultrasonicated for 10 min at maximum intensity. The remaining salt in each sample was pelleted through centrifugation at 21.1 × *g* for 2 min at 4°C. One hundred microliters was transferred to a GC-MS vial for analysis. The full proposed method is publicly available at https://www.protocols.io/view/salty-sample-derivatization-protocol-for-gc-ms-nyxdfxn.

### GC-MS data acquisition.

All derivatized samples were analyzed on an Agilent 7890B GC coupled to an Agilent 5977A single quadrupole mass selective detector. Using an Agilent 7693 autosampler, 1 μl was injected in splitless mode through a GC inlet liner (ultra inert, splitless, single taper, glass wool; Agilent) onto a DB-5MS column (30 m by 0.25 mm, 0.25-μm film thickness, including 10 m DuraGuard column; Agilent). The inlet liner was changed every 50 samples to avoid damage to the GC column and associated shifts in retention times. The injector temperature was set at 290°C. Chromatography was achieved with an initial column oven temperature set at 60°C, followed by a ramp of 20°C min^−1^ until 325°C, and then held for 2 min. Helium carrier gas was used at a constant flow rate of 1 ml min^−1^. Mass spectra were acquired in electron ionization mode at 70 eV across the mass range of *m/z* 50 to 600 and a scan rate of 2 scans s^−1^. The retention time for the method was locked using standard mixture of fatty acid methyl esters (Sigma-Aldrich).

### Data processing and analysis.

Raw Agilent data files were converted to mzXML files using msConvert (46) and imported into XCMS (v. 2.99.6) (47) within the R software environment (v. 3.4.2) for data processing and analysis. Total ion chromatograms (TIC) were obtained using the xcmsRaw function. TICs comparing sample preparation steps were expressed as a percentage of the MQ control. For environmental and cell culture GC-MS profiles, peaks were picked using the matchedFilter algorithm in XCMS with a full width at half maximum set to 8.4, signal to noise threshold at 1, *m/z* width of 0.25 (step parameter), and *m/z* difference between overlapping peaks at 1 (Text S1). The resulting peaks were grouped, and retention times were corrected and regrouped using the density (bandwidth parameter set to 2) and obiwarp methods. Following peak filling, the CAMERA package (v.1.32.0) (48) was used to place *m/z* peaks into pseudospectra by grouping similar peaks with the groupFWHM function. Masses below *m/z* 150 were removed from the resulting peak table to reduce the spectral complexity of noninformative peaks, and all profiles were normalized to the ribitol internal standard. Peaks occurring in blank samples between runs and those with higher relative standard deviation (RSD) scores (>25%) in quality control samples (cell culture experiment only) were removed from the data set. To determine differences in metabolite abundances between sediment habitats, metabolite peak data were analyzed using a Bray-Curtis informed nonmetric multidimensional scaling analysis, followed by an analysis of variance using distance matrices (ADONIS) to test if there are significant differences in metabolite compositions between sites. To identify individual peaks that differed significantly between sediment habitats and between cell culture sampling time points, the resulting peak tables were also log transformed and compared using a one-way analysis of variance. All *P* values were adjusted using the Benjamini-Hochberg (B.H.) method to control for false positives (49). Significant variables exhibiting large fold change differences between starting and ending conditions were further investigated. CAMERA grouped peaks from the environmental survey, and those important to shifts in the cell culture experiment were identified using AMDIS (50). Peaks with NIST hits below 800 were compared to the online data repositories BinVestigate (44) and Golm (43) using the calculated Kovats retention indices (51) based on a reference n-alkane standard (C_7_-C_40_ Saturated Alkanes Standards; Sigma-Aldrich). If no hit was provided, these were considered unknowns.

10.1128/mSystems.00638-19.10 TEXT S1 R script for peak picking for GC-MS data. Download Text S1, PDF file, 0.1 MB. Copyright © 2019 Sogin et al. 2019 Sogin et al. This content is distributed under the terms of the Creative Commons Attribution 4.0 International license.

### Assessing method performance.

To assess the performance of SeaMet, calibration curves using a standard metabolite mix were prepared in ultrapure water, artificial seawater (ASW), and natural seawater from the North Sea (NSW). Curves were calculated from select compounds representing the range of classes SeaMet covers.

To test the performance of SeaMet under a range of organic carbon conditions, samples were prepared for a metabolite recovery experiment by drying down replicate aliquots (*n* = 5) of Marine broth (from the cell culture experiment detailed below) diluted in NSW in different concentrations (0 μl, 50 μl, and 250 μl). Metabolite recovery was assessed by adding 2.5 nmol of the metabolite in the mix to the solution and calculating the variation in detection (% CV) for each compound.

Marine DOM is partly composed of complex compounds including complex sugars and proteins. To show that SeaMet sample preparation does not break apart these compounds, thereby changing the GC-MS signature of a natural sample, biologically relevant concentrations of laminarin (0.2 mg/liter [52]) and bovine serum albumin (BSA) as a proxy for dissolved protein in the oceans (0.67 mg/ml [53]) were dissolved in 0.5 ml of ASW. Replicate samples (*n* = 3) were either prepared with SeaMet or, to simulate harsher conditions during sample preparation, dried using the speed vacuum concentration at 60°C for 16 h. Experimental profiles were compared using a cloud plot.

### Effects of salt and water on metabolite detection.

To test the effect of salt on metabolite derivatization, metabolite mix aliquots were resuspended in 1 ml of ASW ranging in salinity from 0 to 34‰ and dried as described above. Methoxamine-trimethylsilylation (TMS) two-step derivatization was performed by resuspending each sample in 80 μl of MeOX in pyridine (20 mg ml^−1^) and incubating for 90 min at 37°C using a thermal rotating incubator under constant rotation at 1,350 rpm. MSTFA was subsequently added to the mixture, and the mixture was incubated under the same conditions for 90 min (30). Derivatized samples were centrifuged to pellet salt, and the supernatant was transferred to a GC-MS vial for analysis. To test the independent effect of water on metabolite derivatization reactions, MQ water was added to the dried-mixture aliquots in steps of 1 μl from 0 to 10 μl. Replicate water gradient samples were subsequently derivatized as described before using MeOX and MSTFA or by replacing the MSTFA reagent with BSTFA.

### Marine metabolomics method development.

To show how each method development step increased signal intensity and reduced variation in metabolite detection, replicate metabolite mixture aliquots (*n *= 5) were resuspended in 0.5 ml of ASW. Metabolite mixture aliquots (*n *= 5) were also resuspended in MQ water as a no-salt control to highlight the effects of salt and water on metabolite derivatization. Forty microliters of ribitol (0.2 mM) and 100 μl cholestane (1 mM) were added to each aliquot as internal standards. MQ water and ASW samples were first derivatized following the (i) two-step TMS previously described. Successive steps in the proposed protocol were then applied to ASW samples to demonstrate the combined effects on metabolite detection, as follows: (ii) exchange of MSTFA for BSTFA, (iii) removal of residual water from the salt pellet by increasing the speed vacuum drying time and by introducing a toluene drying step to help extract water from the salt pellet, (iv) ultrasonication of the samples after the steps involving the addition of toluene, MeOX, and BSTFA and following the last derivatization step, and (v) drying the MeOX in pyridine reagent between derivatization reactions. The resulting GC-MS profiles were used to show increases in total signals detected with successive changes in the proposed protocol. Additionally, a cloud plot (using processed peak integration data) was generated to compare compounds dissolved in seawater and to show which metabolite ions exhibited significant (B.H. adjusted *P < *0.05) and large fold changes [log_2_(FC), >2] between the standard method and the SeaMet method.

### Solid-phase extraction.

Replicate metabolite mixture aliquots (*n = *6) were resuspended in 2 ml of ASW. Half a milliliter was reserved from each sample to compare GC-MS profiles before and after SPE sample concentration. Inorganic salts were eluted and metabolites extracted from the remaining 1.5-ml mixture following an SPE-based technique using Bond Elut styrene-divinylbenzene (PPL, 100 mg, 3 ml) columns (17). Briefly, the PPL-SPE cartridges were conditioned with 1 column volume (cv) of methanol before 1 ml of the mixture sample was acidified to pH 2 using HCl and loaded onto the column under low vacuum pressure so the flow rate did not exceed 40 ml min^−1^. The column was washed using 0.01 M HCl (2 cv) and allowed to air dry for 5 min. The samples were eluted using 1 ml of methanol. The internal standards ribitol and cholestane were added to both the PPL-SPE-eluted sample (after SPE) and the remaining 1 ml of the metabolite mixture (before SPE). All samples were dried to completeness using a speed vacuum concentrator and prepared for GC-MS analysis using the proposed method for marine metabolomics. The resulting profiles were compared using a cloud plot to show which metabolite ions exhibited significant (B.H. adjusted *P < *0.05) and large fold changes [log_2_(FC), >2] between the before- and after-SPE treatments.

### Cell culture sampling.

Replicate cultures (*n *= 3) *of* Marinobacter adhaerens HP15 DsRed-wild type were cultivated in Marine broth (consisting of 2 g of peptone, 0.4 g of yeast extract, 0.04 g of iron phosphate, 300 ml of filtered and autoclaved North Sea seawater, 100 ml of demineralized water, and 4.8 g of agar) medium at 18°C and 240 rpm, as previously described (34). Medium samples from the cell cultures and a no-bacteria control medium were collected at 0, 4, 8, 12, and 22 h post-culture inoculation. Cell counts were monitored at each time point by measuring the optical density at 600 nm (OD_600_). Sampling was carried out by collecting 2 ml of each culture and pelleting the cells through centrifugation for 10 min at 21.1 × *g* and 4°C. The supernatant was immediately stored at –20°C until preparation for GC-MS analysis. Prior to sample derivatization using SeaMet, ribitol (0.2 mM; 40 μl) and cholestane (100 mM; 100 μl) were added to 0.5 ml of each experimental sample and subsequently dried down in a speed vacuum concentrator (8 h 45°C, VA-Q). To control for technical variation, quality control (QC) samples (*n *= 3) were prepared by combining 0.25 μl of each culture supernatant and an extraction blank generated by drying down 0.5 ml of MQ.

### Environmental sampling.

Replicate porewater profiles were collected from coralline (*n *= 4) and mangrove (*n *= 6) sediments from Carrie Bow Cay (16°04′59″N, 88°04′55″W) and Twin Cayes (16°50′3″N, 88°6′23″W), Belize, using a 1-m steel lance with a 2-μm inner diameter covered by a 0.063-mm steel mesh. Samples (2 ml water) were collected every 5 cm from the sediment surface to 15 cm depth. Samples were immediately frozen at –20°C until further analysis. Directly before preparation for GC-MS, the internal standards ribitol and cholestane were added to 0.5 ml of each environmental sample. The mixture was subsequently prepared for GC-MS analysis using the SeaMet method described above.

### Data availability.

All metabolite profile data are publicly available at MetaboLights (https://www.ebi.ac.uk/metabolights/) under identification numbers MTBLS826, MTBLS839, MTBLS843, MTBLS844, MTBLS848, and MTBLS849.
