# Supplementary material for: Marine Metabolomics: a Method for Nontargeted Measurement of Metabolites in Seawater by Gas Chromatography–Mass Spectrometry
Source: mSystems. 2019 Dec 10;4(6):e00638-19. doi: 10.1128/mSystems.00638-19 (PMC6906741; doi:10.1128/mSystems.00638-19)
Supplement: TABLE S1 [file mSystems.00638-19-st001.pdf]

Nr. Detected	Metabolite standard	Manufacture company	KEGG ID	BRITE hierarchy/ General category	Retention time (s)	Quant. ion	Qualifying ions	Mixture
1	Alanine	Fluka	C00041	Peptides; Amino acids; Common amino acids	531	218	116; 233; 258	Method development*
	Arginine	Fluka	C00062	Peptides; Amino acids; Common amino acids	Not detected			Method development
2	Aspartic acid	Fluka	C00049	Peptides; Amino acids; Common amino acids	858	232	147; 218; 292	Method development
	Carnitine	Fluka	C00318	Amino acid	Not detected			Method development
	Citric acid	Fluka	C00158	Organic acids; Carboxylic acids; Tricarboxylic acids	Not detected			Method development*#
	Cysteine	Fluka	C00097	Peptides; Amino acids; Common amino acids	Not detected			Method development
3	D-mannose	Biolog	C00159	Carbohydrates; Monosaccharides; Aldoses	1126	203	217; 301	Method development
4	D-ribose	Biolog	C00121	Carbohydrates; Monosaccharides; Aldoses	973	204	217; 147	Method development
	Dimethylsulfoniopropionate		C04022	Organic compound	Not detected			Method development
5	Fructose	Sigma-Aldrich	C00095	Carbohydrates; Monosaccharides; Ketoses	1098	307	147; 217; 277	Method development*#

Nr. Detected	Metabolite standard	Manufacture company	KEGG ID	BRITE hierarchy/ General category	Retention time (s)	Quant. ion	Qualifying ions	Mixture
6	Fumarate	Fluka	C00122	Dicarboxylic acid	727	245	143; 147; 217	Method development
7	Galactose	Sigma-Aldrich	C00124	Carbohydrates; Monosaccharides; Aldoses	1124	319	147; 205; 217	Method development
8	Glucose	AppliChem	C00031	Carbohydrates; Monosaccharides; Aldoses	1120	321	147; 160; 205	Method development*#
9	Glutamic acid	Merck	C00025	Peptides; Amino acids; Common amino acids	929	177	73; 147; 246	Method development*#
10	Glutamine	Merck	C00064	Peptides; Amino acids; Common amino acids	824			Method development *
	Glycerophosphate	Merck	C00093	Organic compound	Not detected			Method development
11	Glycine	Fluka	C00037	Peptides; Amino acids; Common amino acids	689	204	147; 174; 248	Method development*#
	Histidine	Fluka	C00135	Peptides; Amino acids; Common amino acids	Not detected			Method development#
12	Isoleucine	Fluka	C00407	Peptides; Amino acids; Common amino acids	684	232	147; 158; 218	Method development
13	L-phenylalanine	Biolog	C00079	Peptides; Amino acids; Common amino acids	897	221	321; 147	Method development

Nr. Detected	Metabolite standard	Manufacture company	KEGG ID	BRITE hierarchy/ General category	Retention time (s)	Quant. ion	Qualifying ions	Mixture
14	Lactic acid	Fluka	C00186	Organic acids; Carboxylic acids; Hydroxycarboxylic acids	480	193	117; 147; 191	Method development*#
15	Lauric acid	Fluka	C002679	Lipids; Fatty acids; Saturated fatty acids	955	215	117; 201; 257	Method development#
16	Leucine	Biolog	C00123	Peptides; Amino acids; Common amino acids	598	169	125; 95	Method development*#
17	Leucine	Biolog	C00123	Peptides; Amino acids; Common amino acids	660	177	86; 146; 188	Method development
18	Lysine	Fluka	C00047	Peptides; Amino acids; Common amino acids	1127	174	218; 230; 317	Method development*#
19	Maleic acid	Fluka	C01384	Dicarboxylic acid	692	245	73; 147; 215	Method development*
20	Malic acid	Sigma-Aldrich	C00149	Organic acids; Carboxylic acids; Hydroxycarboxylic acids	832	233	147; 245; 307	Method development#
21	Maltose	Sigma-Aldrich	C00208	Carbohydrates; Oligosaccharides; Disaccharides	1550	361	204; 217; 319	Method development
22	Mannitol	Fluka	C00392	Carbohydrates; Monosaccharides; Sugar alcohols	1135	319	147; 205; 217	Method development

Nr. Detected	Metabolite standard	Manufacture company	KEGG ID	BRITE hierarchy/ General category	Retention time (s)	Quant. ion	Qualifying ions	Mixture
23	Methionine	Sigma-Aldrich	C00073	Peptides; Amino acids; Common amino acids	779	221	104; 178; 206	Method development
24	Methionine	Sigma-Aldrich	C00073	Peptides; Amino acids; Common amino acids	860	176	128; 73	Method development
25	Myo-inositol	Fluka	C00137	Carbohydrates; Monosaccharides; Sugar alcohols	1224	305	147; 217; 305	Method development*#
26	NAG		C00140	Carbohydrates; monosaccharides; amino sugar	1223			Method development*
27	Ornithine		C00077	Amino acid	931/1064			Method development*#
28	Oxalic Acid		C00209	Dicarboxylic acid	558			Method development*
29	Proline		C00148	Peptides; Amino acids; Common amino acids	689	216	142; 189; 244	Method development
	Pyruvate		C00022	Organic acid	Not detected			Method development
30	Serine	Fluka	C00065	Peptides; Amino acids; Common amino acids	733	204	73; 116; 132	Method development*#
31	Succinate	Merck	C00042	Organic acids; Carboxylic acids; Dicarboxylic acids	696	262	129; 147; 247	Method development*#

Nr. Detected	Metabolite standard	Manufacture company	KEGG ID	BRITE hierarchy/ General category	Retention time (s)	Quant. ion	Qualifying ions	Mixture
32	Sucrose	Fluka	C00089	Carbohydrates; Oligosaccharides; Disaccharides	1495	361	73; 147; 217	Method development*#
33	Threonine	Biolog	C00188	Peptides; Amino acids; Common amino acids	720	255	147; 219; 320	Method development
34	Threonine	Biolog	C00188	Peptides; Amino acids; Common amino acids	756	291	147; 219; 320	Method development
35	Thymine	Fluka	C00178	Nucleic acids; Bases; Pyrimidine	766	255	113; 147; 270	Method development*#
36	Trehalose	Fluka	C01083	Carbohydrates; Oligosaccharides; Disaccharides	1543	377	147; 191; 217	Method development*#
37	Tryptophan		C00078	Peptides; Amino acids; Common amino acids	1289			Method development
38	Urea	Fluka	C00086	Organic compound	644	189	73; 147; 171	Method development
39	Valine	Sigma-Aldrich	C00183	Peptides; Amino acids; Common amino acids	614	188	130; 146; 174	Method development*#
40	2-deoxy-D-ribose	Biolog	C01801	Carbohydrates; Monosaccharides; Deoxy sugars	898	174	250; 100	Database
41	2-hydroxybenzoi	Biolog	C00805	Organic acid	737	146	103; 73	Database
42	3-methyl	Biolog	NA	Sugar	1086	204	217; 147	Database

Nr. Detected	Metabolite standard	Manufacture company	KEGG ID	BRITE hierarchy/ General category	Retention time (s)	Quant. ion	Qualifying ions	Mixture
43	4-hydroxybenzoi	Biolog	C00156	Organic acid	941	292	219; 189	Database
44	Acetic acid		C00033	Organic acids; Carboxylic acids; Monocarboxylic acids	509	147	73; 137	Database
45	Adenosine	Biolog	C00212	Nucleic acids; Nucleosides; Ribonucleosides	1527	361	204; 217	Database
46	Beta-sitosterol	Biolog	C01753	Lipids; Sterol lipids; Sterols	1176	333	292; 319	Database
47	Capric acid	Biolog	C01571	Lipids; Fatty acids; Saturated fatty acids	823	247	359; 147	Database
48	Cellobiose				Cellobiose			Database
49	Cholesterol	Sigma-Aldrich	C00187	Lipids; Sterol lipids; Sterols	1717	247	129; 329; 443	Database#
50	Citraconic acid	Biolog	C02226	Organic acid	733	195	177; 120	Database
51	Citramalic acid	Biolog	C00851	Organic acid	827	158	260; 68	Database
52	Cysteine	Fluka	C00097	Peptides; Amino acids; Common amino acids	885	220	147; 204; 246	Database
53	D-arabinose	Biolog	C00216	Carbohydrates; Monosaccharides; Aldoses	962	307	217; 189	Database
54	D-arabitol	Biolog	C01904	Sugar alcohol	1001	205	307; 319	Database
55	D-galactonic acid-γ-lactone	Biolog	NA	Sugar	1066	261	217; 160	Database

Nr. Detected	Metabolite standard	Manufacture company	KEGG ID	BRITE hierarchy/ General category	Retention time (s)	Quant. ion	Qualifying ions	Mixture
56	D-glucosamine	Biolog	C00329	Carbohydrates; Monosaccharides; Amino sugars	1141	217	307; 319	Database
57	D-mannosamine	Biolog	C03570	Carbohydrates; Monosaccharides; Deoxy sugars	999	217	319; 205	Database
58	D-melibiose	Biolog	C05402	Carbohydrates; Oligosaccharides; Disaccharides	1764	363	469; 334	Database
59	D-psicose	Biolog	C06568	Carbohydrates; Monosaccharides; Ketoses	1097	307	217; 277	Database
60	D-tagatose	Biolog	C00795	Carbohydrates; Monosaccharides; Ketoses	1110	319	217; 205	Database
61	D-tartaric acid	Biolog	C02107	Organic acid	948	252	281; 296	Database
62	D-xylose	Biolog	C00181	Carbohydrates; Monosaccharides; Aldoses	959	217	307; 103	Database
63	D,L-	Biolog	C04227	Organic compound	1200	333	292; 305	Database
64	D,L-α-amino-N-butyric acid	Biolog	NA	Organic acid	516	177	205; 161	Database
65	Dulcitol	Biolog	C01697	Sugar alcohol	1145	217	252; 233	Database
66	Ergosterol	Biolog	C01694	Lipids; Sterol lipids; Sterols	1784	485	394; 255	Database
67	Ethanolamine	Biolog	C00189	Peptides; Amines; Biogenic amines	690	219	130; 117	Database

Nr. Detected	Metabolite standard	Manufacture company	KEGG ID	BRITE hierarchy/ General category	Retention time (s)	Quant. ion	Qualifying ions	Mixture
68	Gentiobiose	Biolog	C08240	Carbohydrates; Oligosaccharides; Disaccharides	1593	361	345; 217	Database
69	Gly-Glu	Biolog	NA	Dipeptide	1193	267	174; 426	Database
70	Glycolic acid	Biolog	C00160	Organic acid	528	174	202; 116	Database
71	Hydroxy-L-proline	Biolog	C01157	Peptides; Amino acids; Other amino acids	849	307	205; 217	Database
72	I-Erythritol	Biolog	C00503	Sugar alcohol	869	304	174; 147	Database
73	Itaconic acid	Biolog	C00490	Dicarboxylic acid	729	147	259; 73	Database
74	L-arabinose	Biolog	C00259	Carbohydrates; Monosaccharides; Aldoses	969	217	307; 277	Database
75	L-arabitol	Biolog	C00532	Sugar alcohol	1002	319	307; 205	Database
76	L-fucose	Biolog	C01019	Carbohydrates; Monosaccharides; Deoxy sugars	1015	174	214; 200	Database
77	L-homoserine	Biolog	C00263	Peptides; Amino acids; Other amino acids	816	229	117; 129	Database
78	L-lyxose	Biolog	C01508	Carbohydrates; Monosaccharides; Aldoses	956	217	307; 189	Database
79	L-rhamnose	Biolog	C00507	Carbohydrates; Monosaccharides; Deoxy sugars	1032	189	217; 147	Database
80	L-sorbose	Biolog	C00247	Carbohydrates; Monosaccharides; Ketoses	1113	204	217; 147	Database

Nr. Detected	Metabolite standard	Manufacture company	KEGG ID	BRITE hierarchy/ General category	Retention time (s)	Quant. ion	Qualifying ions	Mixture
81	Lactitol	Biolog	NA	Sugar alochol	1584	361	204. 217	Database
82	Lactulose	Biolog	C07064	Sugar	1553	307	361; 217	Database
83	m-hydroxyphenylacetic acid	Biolog	NA	Organic acid	939	174	318; 200	Database
84	Maltitol	Biolog	NA	Sugar alochol	1596	361	217; 204	Database
85	Mucic acid	Biolog	C00879	Organic acid	1217	378	319; 246	Database
86	N-acetyl-D-galactosamine	Biolog	C01132	Carbohydrates; Monosaccharides; Amino sugars	1505	230	245; 217	Database
87	N-acetyl-D-glucosamine	Biolog	C00140	Carbohydrates; Monosaccharides; Amino sugars	1234	319	205; 333	Database
88	N-acetyl-D-glucosaminitol	Biolog	C00140	Carbohydrates; Monosaccharides; Amino sugars	1220	319	205; 202	Database
89	N-acetyl-β-D-gannosamine	Biolog	NA	Sugar	1221	205	319; 274	Database
90	N-butylamine	Biolog	C18706	Organic compound	555	205	190; 233	Database
91	Ornithine	Merck	C00077	Peptides; Amino acids; Other amino	1061	162	73; 142; 174	Database

Nr. Detected	Metabolite standard	Manufacture company	KEGG ID	BRITE hierarchy/ General category	Retention time (s)	Quant. ion	Qualifying ions	Mixture
92	p-hydroxyphenylacetic Acid	Biolog	NA	Organic acid	949	217	147; 307	Database
93	Palatinose	Biolog	C01742	Carbohydrates; Oligosaccharides; Disaccharides	1603	361	204; 217	Database
94	Phenylethyl-amine	Biolog	C05332	Peptides; Amines; Biogenic amines	899	247	203; 147	Database
95	Propionic acid		C00163	Organic acids; Carboxylic acids; Monocarboxylic acids	497	147	117; 191	Database
96	Putrescine	Biolog	C00134	Peptides; Amines; Biogenic amines	1017	117	133; 160	Database
97	Sebacic acid	Biolog	C08277	Lipids; Fatty acyls; Fatty acids and conjugates; Dicarboxylic acids	1116	319	205; 217	Database
98	Sorbic acid	Biolog	NA	Organic acid	665	174	147; 100	Database
99	Sorbitol	Merck	C00794	Carbohydrates; Monosaccharides; Sugar alcohols	1134	319	205; 217; 307	Database
100	Stigmasterol	Biolog	C05442	Lipids; Sterol lipids; Sterols	1814	357	396; 487	Database
101	Turanose	Biolog	C19636	Carbohydrates; Oligosaccharides; Disaccharides	1561	361	345; 319	Database

Nr. Detected	Metabolite standard	Manufacture company	KEGG ID	BRITE hierarchy/ General category	Retention time (s)	Quant. ion	Qualifying ions	Mixture
102	Uracil	Biolog	C00106	Nucleic acids; Bases; Pyrimidine	724	259	215; 147	Database
103	α-D-Lactose	Biolog	C00243	Carbohydrates; Oligosaccharides; Disaccharides	1530	361	217; 204	Database
104	α-hydroxybutyri	Biolog	NA	Organic acid	578	191	233; 147	Database
105	α-hydroxyglutari	Biolog	NA	Organic acid	927	296	281; 164	Database
106	α-methyl-D-galactoside	Biolog	NA	Sugar	1095	307	217; 103	Database
107	β-D-allose	Biolog	NA	Aldohexose sugar	1111	205	189; 147	Database
108	β-hydroxybutyric acid	Biolog	C01089	Organic acids; Carboxylic acids; Hydroxycarboxylic acids	580	86	188; 75	Database
109	β-methyl-D-glucoside	Biolog	NA	Sugar	1113	331	215; 129	Database
110	β-methyl-D-glucuronic	Biolog	NA	Sugar	1173	292	305; 319	Database
111	β-methyl-D-xyloside	Biolog	NA	Sugar	997	205	319; 147	Database
112	γ-aminobutyric acid	Biolog	C00334	Peptides; Amino acids; Other amino acids	897	120	146; 91	Database

Nr. Detected	Metabolite standard	Manufacture company	KEGG ID	BRITE hierarchy/ General category	Retention time (s)	Quant. ion	Qualifying ions	Mixture
113	δ-amino-N-valeric acid	Biolog	C00803	Organic acid; Carboxylic acid; Monocarboxylic acids/ ALT: Lipids; Fatty acyls; Fatty acid sand conjugates; Straight chain fatty acids	941	267	223; 193	Database
	*Salt-water							
	# Detection limits							
