# Supplementary figures and images for: Marine Metabolomics: a Method for Nontargeted Measurement of Metabolites in Seawater by Gas Chromatography–Mass Spectrometry
Source: mSystems. 2019 Dec 10;4(6):e00638-19. doi: 10.1128/mSystems.00638-19 (PMC6906741; doi:10.1128/mSystems.00638-19)

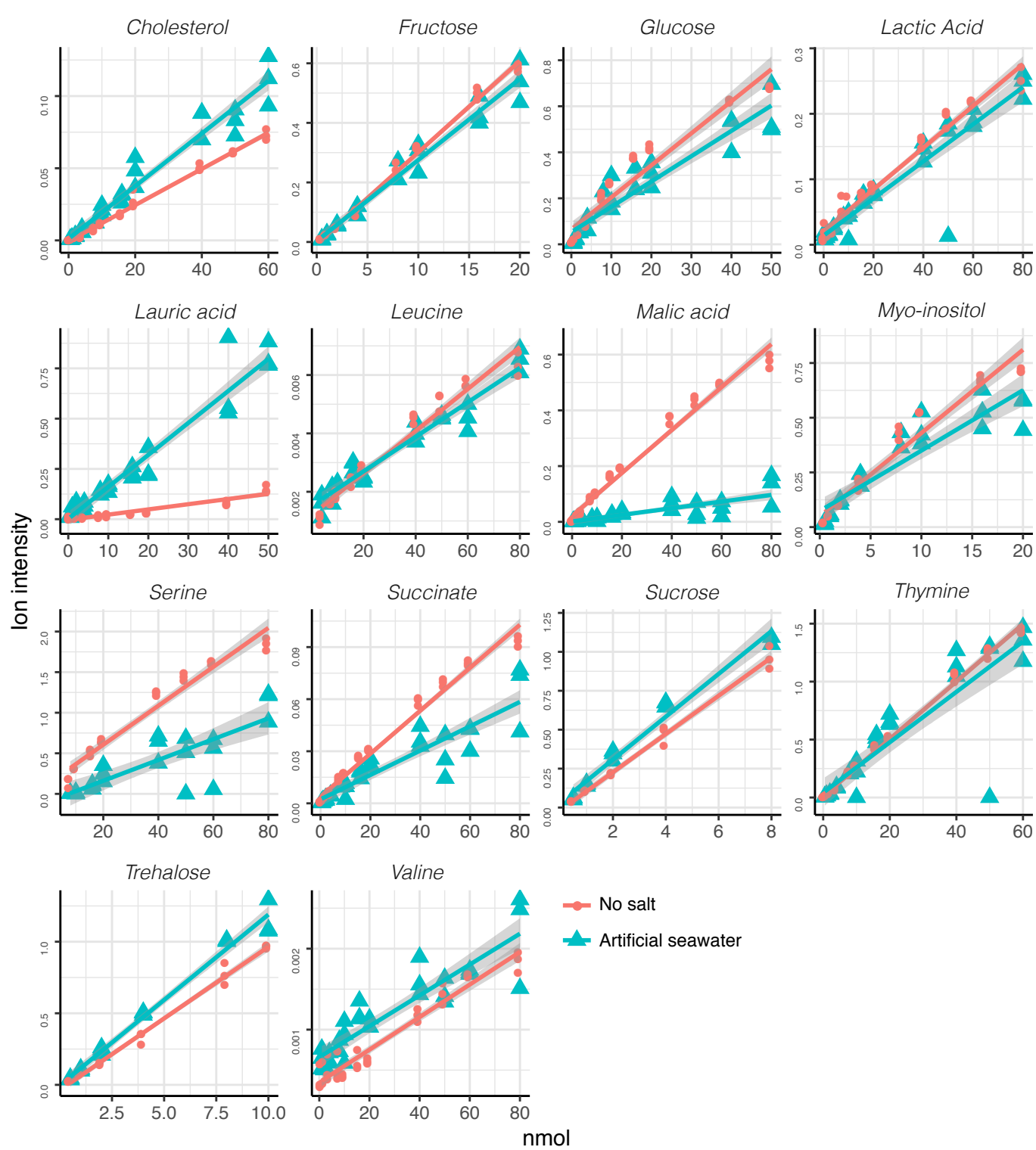

Supplement: FIG S2 [file mSystems.00638-19-sf002.pdf]

**A**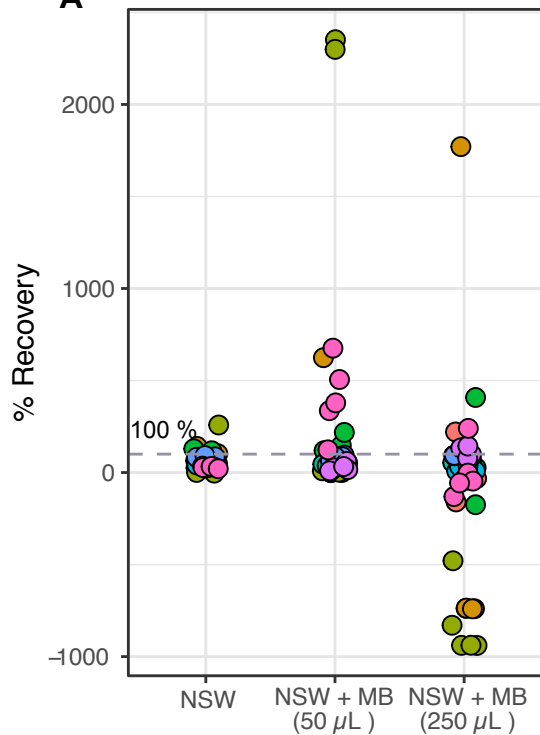**B**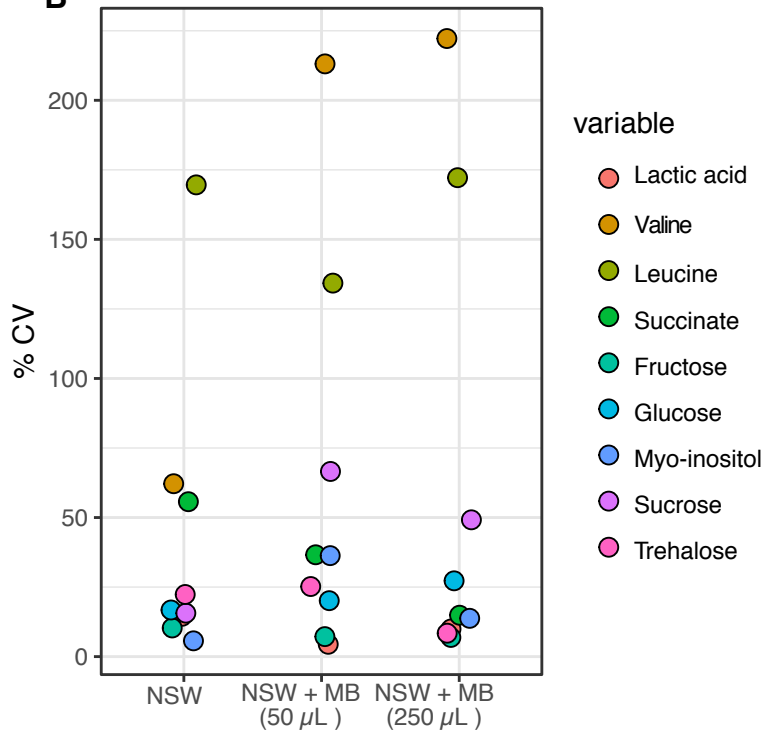

Supplement: FIG S4 [file mSystems.00638-19-sf004.pdf]

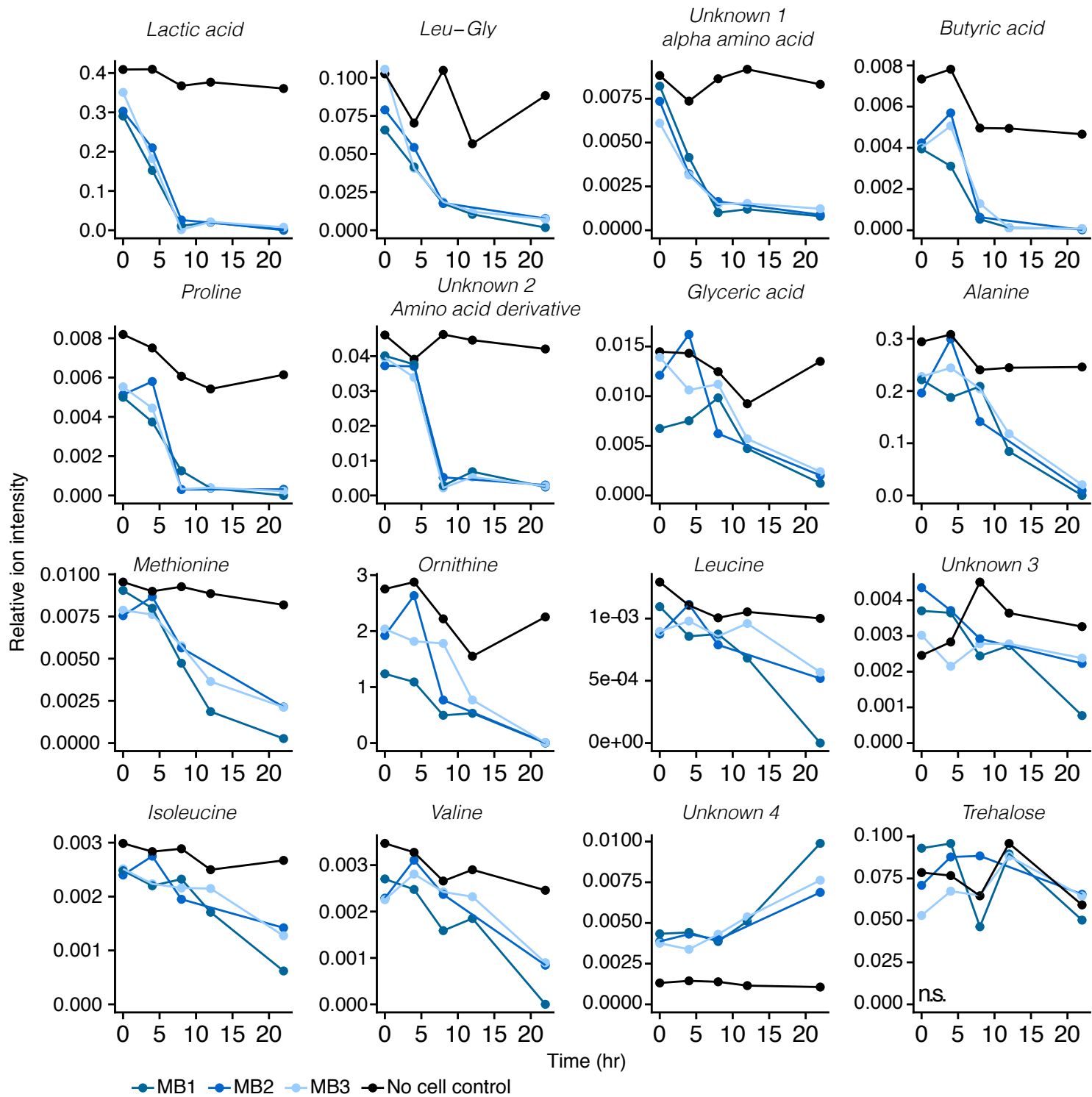

Supplement: FIG S5 [file mSystems.00638-19-sf005.pdf]
