# Supplementary material for: Marine Metabolomics: a Method for Nontargeted Measurement of Metabolites in Seawater by Gas Chromatography–Mass Spectrometry
Source: mSystems. 2019 Dec 10;4(6):e00638-19. doi: 10.1128/mSystems.00638-19 (PMC6906741; doi:10.1128/mSystems.00638-19)
Supplement: TABLE S3 [file mSystems.00638-19-st003.pdf]

**Compound class**	**Sample pre-treatment**	**Detection method**	**Limit of detection**	**Amount of sample required**	**Ref**
Amino acids	o-phthaldialdehyde/ 2-mercaptoethanol derivatization	High performance liquid chromatography	fM	25 µL	**1**
Sugars: Monosaccharides and disaccharides	Acid hydrolysis, desalting	High performance anion exchange chromatography-pulsed amperometric detector	nM	~12 mL	**2**
Sterols	Derivatization	GC-MS	nM	1 L	**3**
	Solid phase extraction	LC/ESI-MS/MS	pM	5 L	**4**

Fatty acids	Solvent extraction	GC-MS	µM	90 L	**5, 6**
Organic acids	Derivatization with 2-nitrophenyl hydrazine and carbodiimine	High pressure liquid chromatography with ion pairing	nM	2 mL	**7**

1 Lindroth P & Mopper K (1979) High performance liquid chromatographic determination of subpicomole amounts of amino acids by precolumn fluorescence derivatization with o-Phthaldialdehyde. *Anal Chem* 51(11):1667-1674.

2 Engel A & Händel N (2011) A novel protocol for determining the concentration and composition of sugars in particulate and in high molecular weight dissolved organic matter (HMW-DOM) in seawater. *Mar Chem* 127(1-4):180-191.

3 Rocha MJ, Cruzeiro C, & Rocha E (2013) Development and validation of a GC-MS method for the evaluation of 17 endocrine disruptor compounds, including phytoestrogens and sitosterol, in coastal waters - their spatial and seasonal levels in Porto costal region (Portugal). *J Water Health* 11(2):281-296.

4 Ronan JM & McHugh B (2013) A sensitive liquid chromatography/tandem mass spectrometry method for the determination of natural and synthetic steroid estrogens in seawater and marine biota, with a focus on proposed Water Framework Directive Environmental Quality Standards. *Rapid Commun Mass Spectrom* 27(7):738-746.

5 Kennicutt MC & Jeffrey LM (1981) Chemical and GC-MS characterization of marine dissolved lipids. *Mar Chem* 10:367-387.

6 Mannino A & Harvey HR (1999) Lipid composition in particulate and dissolved organic matter in the Delaware Estuary: Sources and diagenetic patterns. *Geochimica et Cosmochimica Acta* 63(15):2219-2235.

7 Albert DB & Martens CS (1997) Determination of low-molecular-weight organic acid concentrations in seawater and pore-water samples via HPLC *Mar Chem* 56:27-37.
