# Supplementary material for: Marine Metabolomics: a Method for Nontargeted Measurement of Metabolites in Seawater by Gas Chromatography–Mass Spectrometry
Source: mSystems. 2019 Dec 10;4(6):e00638-19. doi: 10.1128/mSystems.00638-19 (PMC6906741; doi:10.1128/mSystems.00638-19)
Supplement: TABLE S4 [file mSystems.00638-19-st004.pdf]

	A	B	C	D	E	F	G
1	**CAS**	**AMDIS Annotation**	**RT (s)**	**Environment**	**Kovats retention index**	**Golm predicted functional groups**	**BinBase splashID**
2	71144359	>Benzaldehyde, 3-fluoro-4,5-dihydroxy-	441	Coraline Sediments			
3	4325853	>Tris(trimethylsilyl)borate	442	Coraline Sediments			
4	81834517	>Cyclopentene, 3-methyl-1-(trimethylsilyloxy)-	445	Coraline Sediments			
5	13368428	(Trimethylsilyl)morpholine	455	Coraline Sediments			
6	108758	>Pyridine, 2,4,6-trimethyl-	456	Coraline Sediments			
7	360952	Trifluoroacetylmorpholine	460	Coraline Sediments			
8	13871891	>Silane, (cyclohexyloxy)trimethyl-	462	Coraline Sediments			
9	EPA-366582	>Tris(trimethylsilyl)carbamate	471	Coraline Sediments			
10	2116907	>N-trimethylsilylaziridine	471	Coraline Sediments			
11	141628	>Tetrasiloxane, decamethyl-	476	Coraline Sediments			
12	94533627	1,2-di-furan-2-yl-ethane-1,2-diamine	481	Coraline Sediments			
13	2672573	Benzenetricarboxylic acid, trimethyl ester	484	Coraline Sediments			
14	141628	>Tetrasiloxane, decamethyl-	489	Coraline Sediments			

	A	B	C	D	E	F	G
1	**CAS**	**AMDIS Annotation**	**RT (s)**	**Environment**	**Kovats retention index**	**Golm predicted functional groups**	**BinBase splashID**
15	EPA-322566	Diethyl(trimethylsilyl)car bamate	495	Coraline Sediments			
16	17596962	>Propanoic acid, 2-[(trimethylsilyl)oxy]-, trimethylsilyl ester	496	Coraline Sediments *			
17	107460	>Disiloxane, hexamethyl-	500	Coraline Sediments			
18	107517	>Trisiloxane, octamethyl-	501	Coraline Sediments			
19	EPA-318498	phenyl-2,5-dihydro-pyrazolo[3,4-d]pyridazin-4-one	504	Coraline Sediments			
20	33581770	[(trimethylsilyl)oxy]-, trimethylsilyl ester	508	Coraline Sediments *			
21	27844071	(trimethylsilyl)-, trimethylsilyl ester	530	Coraline Sediments			
22	74733996	Methoxyethoxy]ethoxy-1,3-dioxalane	530	Coraline Sediments			
23	18394042	>n-Butylamine, N,N-bis(trimethylsilyl)	534	Coraline Sediments			
24	83239150	[illegible] trimethylsilyloxy-1-(trimethylsilyloxymethyl) ethyl]-5-[(2-trimethylsilyloxy-1-oxopropyl)-N-	552	Coraline Sediments			
25	33581770	[(trimethylsilyl)oxy]-, trimethylsilyl ester	559	Coraline Sediments			

	A	B	C	D	E	F	G
1	**CAS**	**AMDIS Annotation**	**RT (s)**	**Environment**	**Kovats retention index**	**Golm predicted functional groups**	**BinBase splashID**
26	55162328	>Propanoic acid, 3-[(trimethylsilyl)oxy]-, trimethylsilyl ester	562	Coraline Sediments			
27	55517389	bis[(trimethylsilyl)oxyl]-, trimethylsilyl ester	563	Coraline Sediments			
28	1873887	>1,1,1,3,5,5,5-Heptamethyltrisiloxane	571	Coraline Sediments			
29	141639	>Pentasiloxane, dodecamethyl-	581	Coraline Sediments			
30	141639	>Pentasiloxane, dodecamethyl-	613	Coraline Sediments			
31	EPA-332247	>2-Ketobutyric acid eo-tms	621	Coraline Sediments			
32	141639	>Pentasiloxane, dodecamethyl-	628	Coraline Sediments			
33	62185584	>3,6,9,12-Tetraoxa-2,13-disilatetradecane, 2,2,13,13-tetramethyl-	642	Coraline Sediments			
34	1184787	>Methanamine, N,N-dimethyl-, N-oxide	643	Coraline Sediments			
35	75732411	dimethylethyl)dimethyl-, benzoate	650	Coraline Sediments			
36	128649365	>4-Chlorophenyl acridine-9-carboxylate	656	Coraline Sediments			
37	55606741	bis[(trimethylsilyl)oxy]phenyl]ethyl]-1,1,1-trimethyl-N-	663	Coraline Sediments			

	A	B	C	D	E	F	G
1	**CAS**	**AMDIS Annotation**	**RT (s)**	**Environment**	**Kovats retention index**	**Golm predicted functional groups**	**BinBase splashID**
38	6787106	>Glycerol, tris(trimethylsilyl) ether	666	Coraline Sediments *			
39	56484516	>2-Ethyl-2-phenyl-1,3-benzodioxole	667	Coraline Sediments			
40	4282422	>Nonane, 1-iodo-	674	Coraline Sediments			
41	EPA-344400	bromo-4-fluorophenyl heptyl ester	689	Coraline Sediments			
42	5630820	bis(trimethylsilyl)-, trimethylsilyl ester	695	Coraline Sediments			
43	40309577	>Butanedioic acid, bis(trimethylsilyl) ester	699	Coraline Sediments *			
44	38166006	methyl-2,3-bis[(trimethylsilyl)oxy]-, trimethylsilyl ester	703	Coraline Sediments*			
45	55557261	methyl-, bis(trimethylsilyl) ester	709	Coraline Sediments			
46	EPA-315373	>Phthalic acid, di(3-methylphenyl) ester	724	Coraline Sediments			
47	625558	>Formic acid, 1-methylethyl ester	791	Coraline Sediments			
48	71167538	dimethoxycarbonyl-N-methylisoxazolidine	804	Coraline Sediments			
49	19693755	>2-Methoxy-1,3-dioxolane	823	Coraline Sediments			
50	EPA-352084	Ethoxyethoxy)ethoxy]ethoxy-trimethylsilane	846	Coraline Sediments			

	A	B	C	D	E	F	G
1	**CAS**	**AMDIS Annotation**	**RT (s)**	**Environment**	**Kovats retention index**	**Golm predicted functional groups**	**BinBase splashID**
51	EPA-226776	>Bis(ethylthio) difluoromethane	849	Coraline Sediments			
52	10416736	>Trimethyl(2,6 ditert.-butylphenoxy)silane	876	Coraline Sediments			
53	25523142	>3,3-Dichloropropyne	930	Coraline Sediments			
54	38316813	[illegible] (diphenylphosphino)phenyl-C,P]bis(triphenylphosphine)-, (SP-4-2)-	936	Coraline Sediments			
55	35223	pentakis(trimethylsilyl) ether	1003	Coraline Sediments			
56	64096817	>Propanoic acid, 2-acetylhydrazono-	1021	Coraline Sediments			
57	62185584	>3,6,9,12-Tetraoxa-2,13-disilatetradecane, 2,2,13,13-tetramethyl-	1028	Coraline Sediments			
58	EPA-380115	>D-(-)-Ribofuranose, tetrakis(trimethylsilyl) ether (isomer 1)	1032	Coraline Sediments			
59	49647394	bis[3-phenylpropyl]pteridine	1048	Coraline Sediments*			
60	55517389	bis[(trimethylsilyl)oxyl]-, trimethylsilyl ester	1074	Coraline Sediments			
61	18603173	>Tetradecanoic acid, trimethylsilyl ester	1085	Coraline Sediments			

	A	B	C	D	E	F	G
1	**CAS**	**AMDIS Annotation**	**RT (s)**	**Environment**	**Kovats retention index**	**Golm predicted functional groups**	**BinBase splashID**
62	EPA-380134	pentakis(trimethylsilyl) ether	1092	Coraline Sediments*			
63	56196146	pentakis-O-(trimethylsilyl)-, O-	1102	Coraline Sediments*			
64		Galactose	1112	Coraline Sediments*			
65	14317078	hexakis-O-(trimethylsilyl)-	1133	Coraline Sediments			
66	14317078	hexakis-O-(trimethylsilyl)-	1138	Coraline Sediments*			
67	EPA-283555	(4-trimethoxysilyloxy)propa	1145	Coraline Sediments			
68	62185584	>3,6,9,12-Tetraoxa-2,13-disilatetradecane, 2,2,13,13-tetramethyl-	1187	Coraline Sediments			
69	55520893	>Hexadecanoic acid, trimethylsilyl ester	1204	Coraline Sediments			
70	75763	>Silane, tetramethyl-	1243	Coraline Sediments			
71	EPA-363741	diethylhexyloxytridecyloxy-	1300	Coraline Sediments			
72	18748919	>Octadecanoic acid, trimethylsilyl ester	1313	Coraline Sediments			
73	1825634	>Silane, trimethylpropoxy-	1329	Coraline Sediments			
74	EPA-363956	diethylheptyloxytridecyloxy-	1480	Coraline Sediments			

	A	B	C	D	E	F	G
1	**CAS**	**AMDIS Annotation**	**RT (s)**	**Environment**	**Kovats retention index**	**Golm predicted functional groups**	**BinBase splashID**
75	39789134	>5-Nonanol, trimethylsilyl ether	1486	Coraline Sediments			
76	EPA-380091	octakis(trimethylsilyl) ether	1504	Coraline Sediments*			
77		octakis(trimethylsilyl) ether	1553	Coraline Sediments*			
78	EPA-363093	diethylnonyloxytridecyloxy-	1568	Coraline Sediments			
79	481210	>Cholestane	1618	Coraline Sediments			
80	EPA-363960	diethylheptyloxyoctadecyloxy-	1833	Coraline Sediments			
81		Unknown 893	895	Coraline Sediments*	1563	No hits	No hits
82		Unknown 1031	1031	Coraline Sediments*	1759	Primary Alcohol; Secondary alcohol; alcohol; 1,2 diol; Hydroxy	splash10-0fvj-0920000000-2b50a374508a5be92557
83		Unknown 1073	1073	Coraline Sediments*	1823	Hydroxy; Alcohol; Carboylic acid	No hits
84	81834517	>Cyclopentene, 3-methyl-1-(trimethylsilyloxy)-	443	Mangrove sediments			
85	13368428	(Trimethylsilyl)morpholine	453	Mangrove sediments			
86	108758	>Pyridine, 2,4,6-trimethyl-	454	Mangrove sediments			

	A	B	C	D	E	F	G
1	**CAS**	**AMDIS Annotation**	**RT (s)**	**Environment**	**Kovats retention index**	**Golm predicted functional groups**	**BinBase splashID**
87	360952	Trifluoroacetylmorpholine	458	Mangrove sediments			
88	13871891	>Silane, (cyclohexyloxy)trimethyl-	460	Mangrove sediments			
89	EPA-366582	>Tris(trimethylsilyl)carbamate	469	Mangrove sediments			
90	7331842	trimethyl-N-propyl-N-(trimethylsilyl)-	470	Mangrove sediments			
91	141628	>Tetrasiloxane, decamethyl-	474	Mangrove sediments			
92	67370836	Diazacyclopentadecan-6-one, 1-acetyl-	479	Mangrove sediments			
93	141628	>Tetrasiloxane, decamethyl-	488	Mangrove sediments			
94	58792522	>5-Propoxy-2,2'-bipyridyl	490	Mangrove sediments			
95	EPA-322566	Diethyl(trimethylsilyl)carbamate	493	Mangrove sediments			
96	17596962	>Propanoic acid, 2-[(trimethylsilyl)oxy]-, trimethylsilyl ester	494	Mangrove sediments*			
97	EPA-378915	methylenedioxyphenyl]-3-buten-2-one	498	Mangrove sediments			
98	107517	>Trisiloxane, octamethyl-	500	Mangrove sediments			
99	107460	>Disiloxane, hexamethyl-	506	Mangrove sediments			

	A	B	C	D	E	F	G
1	**CAS**	**AMDIS Annotation**	**RT (s)**	**Environment**	**Kovats retention index**	**Golm predicted functional groups**	**BinBase splashID**
100	EPA-329289	ethoxycarbonyl-, dodecyl ester	513	Mangrove sediments*			
101	107460	>Disiloxane, hexamethyl-	550	Mangrove sediments			
102	39981890	>Silanol, trimethyl-, carbonate (2:1)	562	Mangrove sediments			
103	14642796	trimethyl(phenylmethoxy)-	573	Mangrove sediments			
104	141639	>Pentasiloxane, dodecamethyl-	580	Mangrove sediments			
105	EPA-214683	>3,5-Dihydroxy-2H-1,2,4-oxadiazine	591	Mangrove sediments			
106	141639	>Pentasiloxane, dodecamethyl-	612	Mangrove sediments			
107	18394042	>n-Butylamine, N,N-bis(trimethylsilyl)	620	Mangrove sediments			
108	141639	>Pentasiloxane, dodecamethyl-	627	Mangrove sediments			
109	75732411	dimethylethyl)dimethyl-, benzoate	649	Mangrove sediments			
110	EPA-328643	>l-Norvaline, N-(2-methoxyethoxycarbonyl)-, undecyl ester	661	Mangrove sediments			
111	6787106	>Glycerol, tris(trimethylsilyl) ether	665	Mangrove sediments*			
112	63830693	>4-Nonene, 3-methyl-, (Z)-	693	Mangrove sediments			

	A	B	C	D	E	F	G
1	**CAS**	**AMDIS Annotation**	**RT (s)**	**Environment**	**Kovats retention index**	**Golm predicted functional groups**	**BinBase splashID**
113	63830693	>4-Nonene, 3-methyl-, (Z)-	700	Mangrove sediments			
114	4282422	>Nonane, 1-iodo-	709	Mangrove sediments			
115	562492	>Pentane, 3,3-dimethyl-	815	Mangrove sediments			
116	19218941	>Tetradecane, 1-iodo-	840	Mangrove sediments			
117	105577	>Ethane, 1,1-diethoxy-	845	Mangrove sediments			
118	20633130	>Nitric acid, nonyl ester	861	Mangrove sediments			
119	2216355	>2-Bromononane	873	Mangrove sediments			
120	10416736	>Trimethyl(2,6 ditert.-butylphenoxy)silane	875	Mangrove sediments			
121	EPA-309237	>Oxalic acid, allyl nonyl ester	881	Mangrove sediments			
122	7154805	>Heptane, 3,3,5-trimethyl-	888	Mangrove sediments			
123	51764320	>Azetidine, 1,2-dimethyl-	896	Mangrove sediments			
124	562492	>Pentane, 3,3-dimethyl-	903	Mangrove sediments			
125	74381401	methyl-, 1-(1,1-dimethylethyl)-2-methyl-1,3-propanediyl ester	914	Mangrove sediments*			

	A	B	C	D	E	F	G
1	**CAS**	**AMDIS Annotation**	**RT (s)**	**Environment**	**Kovats retention index**	**Golm predicted functional groups**	**BinBase splashID**
126		Lauric acid	937	Mangrove sediments*			
127	834979	>1-Acetyl-4,6,8-trimethylazulene	980	Mangrove sediments			
128	4032864	>Heptane, 3,3-dimethyl-	990	Mangrove sediments			
129	625741	>Propane, 2-methyl-1-nitro-	992	Mangrove sediments			
130	35223	pentakis(trimethylsilyl) ether	1002	Mangrove sediments			
131	EPA-368587	[illegible] (trimethylsilyloxy)-5-((trimethylsilyloxy)methyl)-3,6,10-trioxa-2,11-disiladodecane	1015	Mangrove sediments			
132	EPA-309243	>Oxalic acid, allyl pentadecyl ester	1016	Mangrove sediments			
133	EPA-309202	>Sulfurous acid, 2-ethylhexyl hexyl ester	1019	Mangrove sediments			
134	63830693	>4-Nonene, 3-methyl-, (Z)-	1022	Mangrove sediments			
135	EPA-368588	oxybis(methylene)bis(2,2,7,7-tetramethyl-3,6-dioxa-2,7-disilaoctane)	1026	Mangrove sediments			
136	EPA-368588	oxybis(methylene)bis(2,2,7,7-tetramethyl-3,6-dioxa-2,7-disilaoctane)	1027	Mangrove sediments			

	A	B	C	D	E	F	G
1	**CAS**	**AMDIS Annotation**	**RT (s)**	**Environment**	**Kovats retention index**	**Golm predicted functional groups**	**BinBase splashID**
137	EPA-380115	>D-(-)-Ribofuranose, tetrakis(trimethylsilyl) ether (isomer 1)	1030	Mangrove sediments			
138	6790370	>2-Methyl-3-vinyl-oxirane	1035	Mangrove sediments			
139	49852359	>2-Hepten-4-one, 6-methyl-	1042	Mangrove sediments			
140		Azelaic acid	1048	Mangrove sediments*			
141	EPA-152343	>Borane, diethyl(decyloxy)-	1060	Mangrove sediments			
142	18603173	>Tetradecanoic acid, trimethylsilyl ester	1084	Mangrove sediments			
143	EPA-314901	>Phthalic acid, cyclobutyl hexyl ester	1095	Mangrove sediments			
144	5694688	>1,3-Dioxolane-2-methanol	1109	Mangrove sediments			
145	563166	>Hexane, 3,3-dimethyl-	1115	Mangrove sediments			
146	EPA-283076	>4-Dimethylisopropylsilyloxy hexadecane	1122	Mangrove sediments			
147	82304663	>7,9-Di-tert-butyl-1-oxaspiro(4,5)deca-6,9-diene-2,8-dione	1124	Mangrove sediments			
148	54410989	>1-Nonene, 4,6,8-trimethyl-	1126	Mangrove sediments*			

	A	B	C	D	E	F	G
1	**CAS**	**AMDIS Annotation**	**RT (s)**	**Environment**	**Kovats retention index**	**Golm predicted functional groups**	**BinBase splashID**
149		Mannitol	1138	Mangrove sediments*			
150	74367229	>n-Pentadecanoic acid, trimethylsilyl ester	1144	Mangrove sediments			
151	4160752	>2-Propanone, 1-cyclopropyl-	1149	Mangrove sediments			
152	EPA-309202	>Sulfurous acid, 2-ethylhexyl hexyl ester	1152	Mangrove sediments			
153	20633038	>3,4-Hexanedione, 2,2,5-trimethyl-	1162	Mangrove sediments			
154	1189997	>Heptane, 2,5,5-trimethyl-	1167	Mangrove sediments			
155	EPA-298836	>4-Azaphenanthrene, 1-methyl-3-phenylethynyl-	1176	Mangrove sediments			
156		Palmitic acid	1177	Mangrove sediments*			
157	13427435	>1-Hexene, 3,3,5-trimethyl-	1194	Mangrove sediments			
158	10444072	methoxy-3-methylbenzofuran)	1201	Mangrove sediments			
159	55520893	>Hexadecanoic acid, trimethylsilyl ester	1203	Mangrove sediments			
160		Isooctyl laurate	1235	Mangrove sediments*			
161	104255814	tert-butyldimethylsilyl ester	1242	Mangrove sediments			

	A	B	C	D	E	F	G
1	**CAS**	**AMDIS Annotation**	**RT (s)**	**Environment**	**Kovats retention index**	**Golm predicted functional groups**	**BinBase splashID**
162	55517583	>Heptadecanoic acid, trimethylsilyl ester	1258	Mangrove sediments			
163	122085614	>Phosphine oxide, bis(pentamethylphenyl)-	1264	Mangrove sediments			
164	1116650	bis(trimethylsiloxy)propy l ester	1289	Mangrove sediments*			
165	EPA-363741	diethylhexyloxytridecylox y-	1299	Mangrove sediments			
166	18748919	>Octadecanoic acid, trimethylsilyl ester	1313	Mangrove sediments			
167	EPA-367922	>Formic acid, 2-ethylbutyl ester	1324	Mangrove sediments			
168	94883973	>(1-Propoxy-pentyl)-cyclopropane	1332	Mangrove sediments			
169	87734665	>7H-Benzo[c]furo[2,3-f][1]benzopyran, 2,7,7,10-tetramethyl-4-pentyl-	1336	Mangrove sediments			
170	EPA-363080	>Silane, diethyldecyloxyoctyloxy-	1369	Mangrove sediments			
171	33648769	>2-Deoxy ribose O,O',O''-tris(trimethylsilyl)-	1371	Mangrove sediments*			
172	563166	>Hexane, 3,3-dimethyl-	1381	Mangrove sediments			
173	1188734	bis(trimethylsiloxy)propy l ester	1387	Mangrove sediments*			
174	EPA-315373	>Phthalic acid, di(3-methylphenyl) ester	1391	Mangrove sediments			

	A	B	C	D	E	F	G
1	**CAS**	**AMDIS Annotation**	**RT (s)**	**Environment**	**Kovats retention index**	**Golm predicted functional groups**	**BinBase splashID**
175	EPA-309237	>Oxalic acid, allyl nonyl ester	1411	Mangrove sediments			
176	EPA-363068	diethylisobutoxypentadecyloxy-	1417	Mangrove sediments			
177	EPA-112561	methyl-2-tetrahydrofuryl)tetrahydr	1428	Mangrove sediments			
178	EPA-153631	glycerine-(1)-monoester, bis-O-trimethylsilyl-	1434	Mangrove sediments*			
179	EPA-363956	diethylheptyloxytridecyloxy-	1463	Mangrove sediments			
180	53212978	Monopalmitoylglycerol trimethylsilyl ether	1464	Mangrove sediments*			
181	1689834	>Benzonitrile, 4-hydroxy-3,5-diiodo-	1481	Mangrove sediments*			
182	1188745	bis[(trimethylsilyl)oxy]propyl ester	1482	Mangrove sediments			
183	544763	>Hexadecane	1490	Mangrove sediments			
184		Sucrose	1503	Mangrove sediments*			
185	EPA-153632	glycerine-(1)-monoester, bis-O-trimethylsilyl-	1507	Mangrove sediments*			
186	EPA-153632	glycerine-(1)-monoester, bis-O-trimethylsilyl-	1512	Mangrove sediments			
187	EPA-153632	glycerine-(1)-monoester, bis-O-trimethylsilyl-	1524	Mangrove sediments*			

	A	B	C	D	E	F	G
1	**CAS**	**AMDIS Annotation**	**RT (s)**	**Environment**	**Kovats retention index**	**Golm predicted functional groups**	**BinBase splashID**
188	EPA-309202	>Sulfurous acid, 2-ethylhexyl hexyl ester	1535	Mangrove sediments			
189	EPA-363325	diethylisohexyloxyoctadecyloxy-	1537	Mangrove sediments			
190	53336133	>2-Monostearin trimethylsilyl ether	1551	Mangrove sediments*			
191	EPA-363108	diethylheptadecyloxy(2-methylbutoxy)-	1568	Mangrove sediments			
192	1188756	bis[(trimethylsilyl)oxy]propyl ester	1570	Mangrove sediments*			
193	1188756	bis[(trimethylsilyl)oxy]propyl ester	1571	Mangrove sediments			
194	544763	>Hexadecane	1580	Mangrove sediments			
195	EPA-153824	glycerine-(1)-monoester, bis-O-trimethylsilyl-	1607	Mangrove sediments*			
196	20690839	2',6'-dimethoxy-4,4'-methylenedi-	1617	Mangrove sediments			
197	481210	>Cholestane	1617	Mangrove sediments			
198	1072168	>Octane, 2,7-dimethyl-	1620	Mangrove sediments			
199	55517947	bis[(trimethylsilyl)oxy]propyl ester	1646	Mangrove sediments*			
200	61227870	>2,5-Octadiyne, 4,4-diethyl-	1655	Mangrove sediments			

	A	B	C	D	E	F	G
1	**CAS**	**AMDIS Annotation**	**RT (s)**	**Environment**	**Kovats retention index**	**Golm predicted functional groups**	**BinBase splashID**
201	463821	>Neopentane	1660	Mangrove sediments			
202	EPA-363960	diethylheptyloxyoctadecyloxy-	1685	Mangrove sediments			
203	EPA-363331	diethyldecyloxyhexadecyloxy-	1723	Mangrove sediments			
204	EPA-365944	>2-ethenyl-3-ethylpyrazine	1752	Mangrove sediments			
205	EPA-363972	diethylhexadecyloxyundecyloxy-	1763	Mangrove sediments			
206	EPA-363960	diethylheptyloxyoctadecyloxy-	1830	Mangrove sediments			
207		Unknown 1031	1031	Mangrove sediments*	1759	Primary Alcohol; Secondary alcohol; alcohol; 1,2 diol; Hydroxy	splash10-0fvj-0920000000-2b50a374508a5be92557
208		Unkonwn 1595	1595	Mangrove sediments*	2832	Alcohol	No hits
209		Unknown 1635	1635	Mangrove sediments*	2930	Alcohol; Hydroxy; primary alcohol	no hits
210		Unknown 1672	1672	Mangrove sediments*	2023	Alcohol; Hydroxy; primary alcohol	No hits
211		Unknown 1721	1721	Mangrove sediments*	3151	Hydroxy alcohol; carboxylic acid; primary alcohol	splash10-05o0-1910000000-49e59c211689b393e2a6

	A	B	C	D	E	F	G
1	**CAS**	**AMDIS Annotation**	**RT (s)**	**Environment**	**Kovats retention index**	**Golm predicted functional groups**	**BinBase splashID**
212		Unknown 1734	1734	Mangrove sediments*	3185	Alcohol; Hydroxy primary alcohol	No hits
213		Unkonwn 1746	1746	Mangrove sediments*	3215	Alcohol; hydroxy; Primary alcohol	No hits
214		Unknown 2005	2005	Mangrove sediments*	3691	Alcohol; hydroxy; alkene	No hits
215		Unknown 2035	2035	Mangrove sediments*	NA	Hydroxy alcohol; carboxylic acid; primary alcohol	No hits
216	502976	>1,4-Dioxane-2,5-dione	442	Cell culture			
217	13871891	(cyclohexyloxy)trimethyl-	444	Cell culture			
218	95681	dimethyl-	445	Cell culture			
219	35227840	Biphenylylenephosphoric	450	Cell culture			
220	EPA-366582	>Tris(trimethylsilyl)carbamate	452	Cell culture			
221	7331842	trimethyl-N-propyl-N-(trimethylsilyl)-	453	Cell culture			
222	107460	>Disiloxane, hexamethyl-	457	Cell culture			
223	141628	decamethyl-	459	Cell culture			
224	53274854	bis(trimethylsiloxy)-	465	Cell culture			
225	EPA-147861	benzylidene-coumaran-3-one	466	Cell culture			
226	141628	decamethyl-	474	Cell culture			
227	EPA-314859	>Phthalimide, N-isopropyl-	478	Cell culture			
228	17596962	>Propanoic acid, 2-[(trimethylsilyl)oxy]-, trimethylsilyl ester	480	Cell culture			

	A	B	C	D	E	F	G
1	**CAS**	**AMDIS Annotation**	**RT (s)**	**Environment**	**Kovats retention index**	**Golm predicted functional groups**	**BinBase splashID**
229	17301325	>Undecane, 4,7-dimethyl-	483	Cell culture			
230	18294047	>Ethanedioic acid, bis(trimethylsilyl) ester	484	Cell culture			
231	107517	>Trisiloxane, octamethyl-	485	Cell culture			
232	111433	>Di-n-propyl ether	489	Cell culture			
233	EPA-216941	Pentamethyldisilanyloxy pentane	494	Cell culture			
234	1630768	propyl-	501	Cell culture			
235	52060852	>Propanoic acid, 2-oxo-, trimethylsilyl ester	502	Cell culture			
236	51099559	2H-pyrrole-2-carboxylic acid, ethyl ester	504	Cell culture			
237	7480786	ester	507	Cell culture			
238	EPA-328619	propargyloxycarbonyl-, isohexyl ester	512	Cell culture			
239	563166	>Hexane, 3,3-dimethyl-	515	Cell culture			
240	27844071	(trimethylsilyl)-, trimethylsilyl ester	517	Cell culture			
241	18394042	>n-Butylamine, N,N-bis(trimethylsilyl)	522	Cell culture			
242	93677668	oxybis-	524	Cell culture			
243	1189997	trimethyl-	525	Cell culture			
244	3376327	methyloxime	528	Cell culture			
245	7364423	(trimethylsilyl)-, trimethylsilyl ester	533	Cell culture			
246	40333071	methyl-, bis(trimethylsilyl) ester	540	Cell culture			

	A	B	C	D	E	F	G
1	**CAS**	**AMDIS Annotation**	**RT (s)**	**Environment**	**Kovats retention index**	**Golm predicted functional groups**	**BinBase splashID**
247	EPA-332781	>3-Furoic acid, trimethylsilyl ester	547	Cell culture			
248	EPA-324066	>Terephthalic acid, phenyl octyl ester	548	Cell culture			
249	EPA-353273	2-[(trimethylsilyl)oxy]prop-	549	Cell culture			
250	5501393	acetyl-4-hydroxy-6-methyl-	551	Cell culture			
251	27531493	1,6-dihydro-1,5-dimethyl-6-phenyl-	552	Cell culture			
252	18412685	dimethylethyl)phenyl]trimethyl-	554	Cell culture			
253	14468907	pyrrolidinone	555	Cell culture			
254	14468907	pyrrolidinone	556	Cell culture			
255	EPA-333250	>l-Leucine, trimethylsilyl ester	565	Cell culture			
256	14642796	trimethyl(phenylmethoxy	565	Cell culture			
257	EPA-375246	alcohol, 1-methylpropyl ether	568	Cell culture			
258	141639	dodecamethyl-	570	Cell culture			
259	EPA-332992	N-trimethylsilyl-, trimethylsilyl ester	576	Cell culture			
260	541026	decamethyl-	580	Cell culture			
261	71330	2,4(1H,3H)-dione	589	Cell culture			
262	18294047	>Ethanedioic acid, bis(trimethylsilyl) ester	593	Cell culture			

	A	B	C	D	E	F	G
1	**CAS**	**AMDIS Annotation**	**RT (s)**	**Environment**	**Kovats retention index**	**Golm predicted functional groups**	**BinBase splashID**
263	96555429	d]pyrimidin-4,6(5H,7H)-dione-3-carboxamide	598	Cell culture			
264	141639	dodecamethyl-	603	Cell culture			
265	62016379	>Octane, 2,4,6-trimethyl-	606	Cell culture			
266	EPA-192859	methoxyphenyl)-N,N,N',N'-tetramethylethane-1,2-	613	Cell culture			
267	7364445	(trimethylsilyl)-, trimethylsilyl ester	614	Cell culture			
268	18394042	>n-Butylamine, N,N-bis(trimethylsilyl)	616	Cell culture			
269	55133954	>Butanoic acid, 4-[(trimethylsilyl)oxy]-, trimethylsilyl ester	631	Cell culture			
270	7335173	octanone	633	Cell culture			
271	563166	>Hexane, 3,3-dimethyl-	640	Cell culture			
272	2078128	trimethylsilyl ester	645	Cell culture			
273	1014604	>Benzene, 1,3-bis(1,1-dimethylethyl)-	650	Cell culture			
274	57162740	azafluorene	654	Cell culture			
275	5269374	ester	657	Cell culture			
276	17301325	>Undecane, 4,7-dimethyl-	658	Cell culture			
277	10497059	phosphate (3:1)	660	Cell culture			
278	70125610	>6-Phenylisoquinoline	661	Cell culture			
279	7364467	(trimethylsilyl)-, trimethylsilyl ester	662	Cell culture			
280	56484516	benzodioxole	664	Cell culture			

	A	B	C	D	E	F	G
1	**CAS**	**AMDIS Annotation**	**RT (s)**	**Environment**	**Kovats retention index**	**Golm predicted functional groups**	**BinBase splashID**
281	544763	>Hexadecane	669	Cell culture			
282	33581769	tris(trimethylsiloxy)-	674	Cell culture			
283	17301303	>Undecane, 3,8-dimethyl-	676	Cell culture			
284	33581769	tris(trimethylsiloxy)-	677	Cell culture			
285	7483923	(trimethylsilyl)-, trimethylsilyl ester	679	Cell culture			
286	55771409	nonene	682	Cell culture			
287	7364478	(trimethylsilyl)-, trimethylsilyl ester	684	Cell culture			
288	39538119	>Butanoic acid, 4-[(trimethylsilyl)amino]-, trimethylsilyl ester	687	Cell culture			
289	90744459	>Malonic acid, bis(2-trimethylsilylethyl ester	689	Cell culture			
290	5630820	bis(trimethylsilyl)-, trimethylsilyl ester	690	Cell culture			
291	17312537	>Decane, 3,6-dimethyl-	695	Cell culture			
292	40309577	>Butanedioic acid, bis(trimethylsilyl) ester	697	Cell culture			
293	38166006	methyl-2,3-bis[(trimethylsilyl)oxy]-, trimethylsilyl ester	699	Cell culture			
294	2216344	>Octane, 4-methyl-	704	Cell culture			
295	17312593	>Undecane, 4-ethyl-	707	Cell culture			
296	2003921	Octamethyl-3,5-bis(trimethylsiloxy)tetras	713	Cell culture			

	A	B	C	D	E	F	G
1	**CAS**	**AMDIS Annotation**	**RT (s)**	**Environment**	**Kovats retention index**	**Golm predicted functional groups**	**BinBase splashID**
297	10457144	>Pyrimidine, 2,4-bis[(trimethylsilyl)oxy]-	716	Cell culture			
298	EPA-352979	trimethylsilyl-, trimethylsilyl ester	717	Cell culture			
299	107528	tetradecamethyl-	728	Cell culture			
300	64625178	bis(trimethylsilyl)-, trimethylsilyl ester	733	Cell culture			
301	EPA-320706	ethoxycarbonyl-, nonyl ester	735	Cell culture			
302	82326112	trimethylsilyl ester	737	Cell culture			
303	55255442	acid, 1-(trimethylsilyl)-, trimethylsilyl ester	739	Cell culture			
304	239441	a]isoquinoline	742	Cell culture			
305	107460	>Disiloxane, hexamethyl-	743	Cell culture			
306	261502936	trimethylsilyloxyphenyl)propane	747	Cell culture			
307	32741245	5,8-dihydroxy-2,3,7-trimethyl-	751	Cell culture			
308	7537022	Tris(trimethylsilyl)-L-threonine	753	Cell culture			
309	7537022	Tris(trimethylsilyl)-L-threonine	757	Cell culture			
310	EPA-105079	>3-Methylbutyl N,O-bis(heptafluorobutyryl)hydroxyprolinate	761	Cell culture			
311	EPA-322566	Diethyl(trimethylsilyl)carbamate	769	Cell culture			

	A	B	C	D	E	F	G
1	**CAS**	**AMDIS Annotation**	**RT (s)**	**Environment**	**Kovats retention index**	**Golm predicted functional groups**	**BinBase splashID**
312	55191521	>Butanoic acid, 2,4-bis[(trimethylsilyl)oxy]-, trimethylsilyl ester	774	Cell culture			
313	32383769	>Medicarpin	776	Cell culture			
314	EPA-333294	>l-Methionine, trimethylsilyl ester	778	Cell culture			
315	15985054	(trimethylsilyl)aspartic acid	779	Cell culture			
316	563166	>Hexane, 3,3-dimethyl-	780	Cell culture			
317	EPA-333288	>l-Aspartic acid, bis(trimethylsilyl) ester	786	Cell culture			
318	EPA-329585	ethoxycarbonyl-, decyl ester	788	Cell culture			
319	55191532	>Butanoic acid, 3,4-bis[(trimethylsilyl)oxy]-, trimethylsilyl ester	790	Cell culture			
320	55320111	(trimethylsilyl)-, trimethylsilyl ester	795	Cell culture			
321	53274854	bis(trimethylsiloxy)-	797	Cell culture			
322	EPA-344664	>Isophthalic acid, di(2-fluorophenyl) ester	799	Cell culture			
323	84498475	>Benzoic alcohol, 2-hydroxy-3,5-dinitro-	801	Cell culture			
324	1.177E+09	>L-Homoserine, N,O-bis(trimethylsilyl)-, trimethylsilyl ester	804	Cell culture			

	A	B	C	D	E	F	G
1	**CAS**	**AMDIS Annotation**	**RT (s)**	**Environment**	**Kovats retention index**	**Golm predicted functional groups**	**BinBase splashID**
325	32565121	(trimethylsilyl)-3-[(trimethylsilyl)amino]-	811	Cell culture			
326	104255745	>Decanoic acid, tert-butyldimethylsilyl ester	814	Cell culture			
327	1009934	Hexamethylcyclotrisilaza ne	818	Cell culture			
328	32741245	5,8-dihydroxy-2,3,7-trimethyl-	821	Cell culture			
329	EPA-79453	monoamide, O,O'-bis(trimethylsilyl)-	823	Cell culture			
330	1560970	>Dodecane, 2-methyl-	836	Cell culture			
331	1560969	>Tridecane, 2-methyl-	840	Cell culture			
332	EPA-353273	2-[(trimethylsilyl)oxy]prop-	841	Cell culture			
333	EPA-325648	>Carbonic acid, butyl 2-fluorophenyl ester	843	Cell culture			
334	EPA-330916	>Pivalic acid, 3-fluorophenyl ester	845	Cell culture			
335	74810472	Pyrimidinedione, dihydro-1,3-bis(trimethylsilyl)-	847	Cell culture			
336	55268536	(trimethylsilyl)-, bis(trimethylsilyl) ester	858	Cell culture			
337	27844106	(trimethylsilyl)-, trimethylsilyl ester	859	Cell culture			
338	30274772	(trimethylsilyl)-, trimethylsilyl ester	862	Cell culture			

	A	B	C	D	E	F	G
1	**CAS**	**AMDIS Annotation**	**RT (s)**	**Environment**	**Kovats retention index**	**Golm predicted functional groups**	**BinBase splashID**
339	5630819	trimethyl-N-(trimethylsilyl)-N-[2-[(trimethylsilyl)oxy]ethyl	867	Cell culture			
340	73105676	methylundecane	875	Cell culture			
341	10416736	>Trimethyl(2,6 ditert.-butylphenoxy)silane	876	Cell culture			
342	2899425	>Alanine, phenyl-, trimethylsilyl ester, dl-	883	Cell culture			
343	55517481	>2-Piperidinecarboxylic acid, 1-(trimethylsilyl)-5-[(trimethylsilyl)oxy]-, trimethylsilyl ester	891	Cell culture			
344	27750523	>Acetic acid, [o-(trimethylsiloxy)phenyl]-, trimethylsilyl ester	894	Cell culture			
345	60022879	>Pentanedioic acid, 2-(methoxyimino)-, bis(trimethylsilyl) ester	899	Cell culture			
346	3042215	phenyl-	919	Cell culture			
347	544763	>Hexadecane	922	Cell culture			
348	24595708	>Ornithine, tri-TMS	928	Cell culture			
349	15985076	(trimethylsilyl)-, bis(trimethylsilyl) ester,	931	Cell culture			
350	EPA-215422	dimethylethyl)-4-diphenylmethylenecyclohexa-2,5-dien-1-one	932	Cell culture			

	A	B	C	D	E	F	G
1	**CAS**	**AMDIS Annotation**	**RT (s)**	**Environment**	**Kovats retention index**	**Golm predicted functional groups**	**BinBase splashID**
351	2899527	(trimethylsilyl)phenylalanine	937	Cell culture			
352	2899527	(trimethylsilyl)phenylalanine	938	Cell culture			
353	EPA-214460	>9-[4-[1,3-Diphenyl-2-imidazolidinyl]-2,3-O-[1-methylethylidene]-··-d-	939	Cell culture			
354	EPA-351997	[illegible] Pentafluoropropanoyl)oxyethoxy]ethoxy]ethoxy]ethoxy]ethyl 2,2,3,3,3-pentafluoropropanoate	940	Cell culture			
355	17962037	>2-Butenedioic acid (E)-, bis(trimethylsilyl) ester	960	Cell culture			
356	55649622	>L-Asparagine, N,N2-bis(trimethylsilyl)-, trimethylsilyl ester	965	Cell culture			
357	56196146	pentakis-O-(trimethylsilyl)-, O-	967	Cell culture			
358	107770990	>3,5-Dimethyldodecane	972	Cell culture			
359	3892000	trimethyl-	972	Cell culture			
360	EPA-324069	>Terephthalic acid, phenyl undecyl ester	973	Cell culture			
361	EPA-352398	Tris(hydroxymethyl)propane, tris(trimethylsilyl)	976	Cell culture			
362	76358800	>Silane, (1,1-dimethylethyl)(hexadecyloxy)dimethyl-	977	Cell culture			

	A	B	C	D	E	F	G
1	**CAS**	**AMDIS Annotation**	**RT (s)**	**Environment**	**Kovats retention index**	**Golm predicted functional groups**	**BinBase splashID**
363	68595506	chloro-2-methoxybenzoyl)-4-	984	Cell culture			
364	EPA-333699	trimethylsilyl-, trimethylsilyl ester	985	Cell culture			
365	24595695	bis(trimethylsilyl)-, trimethylsilyl ester	991	Cell culture			
366	20967435	trifluoro-1-trifluoromethylethyl)-4,5-dihydrooxazole	995	Cell culture			
367	35223	pentakis(trimethylsilyl) ether	1000	Cell culture			
368	17556104	homoandrostan-17-one, (5‡)-	1011	Cell culture			
369	EPA-333689	trimethylsilyl-, trimethylsilyl ester	1018	Cell culture			
370	124185	>Decane	1023	Cell culture			
371	EPA-347187	phenylprop-2-yl)phenoxy)propoxy-	1028	Cell culture			
372	EPA-333692	trimethylsilyl-, trimethylsilyl ester	1052	Cell culture			
373	68595846	triTMS	1053	Cell culture			
374	EPA-333692	trimethylsilyl-, trimethylsilyl ester	1056	Cell culture			
375	EPA-333703	trimethylsilyl-, trimethylsilyl ester	1058	Cell culture			

	A	B	C	D	E	F	G
1	**CAS**	**AMDIS Annotation**	**RT (s)**	**Environment**	**Kovats retention index**	**Golm predicted functional groups**	**BinBase splashID**
376	EPA-333139	>DL-Ornithine, N,N,N'-tris(trimethylsilyl)-, trimethylsilyl ester	1062	Cell culture			
377	EPA-333139	>DL-Ornithine, N,N,N'-tris(trimethylsilyl)-, trimethylsilyl ester	1062	Cell culture			
378	EPA-318480	acid, fluoren-9-ylidene-hydrazide	1064	Cell culture			
379	556901	imino-	1070	Cell culture			
380	18406058	>Benzoic acid, 4-[(trimethylsilyl)amino]-, trimethylsilyl ester	1075	Cell culture			
381	EPA-333696	trimethylsilyl-, trimethylsilyl ester	1082	Cell culture			
382	56196146	pentakis-O-(trimethylsilyl)-, O-	1094	Cell culture			
383	17995049	>9H-Purin-6-amine, N,9-bis(trimethylsilyl)-	1097	Cell culture			
384	EPA-328940	methoxycarbonyl-, dodecyl ester	1098	Cell culture			
385	EPA-333692	trimethylsilyl-, trimethylsilyl ester	1100	Cell culture			
386	130405106	pentakis-O-(trimethylsilyl)-, o-methyloxyme, (1E)-	1103	Cell culture			

	A	B	C	D	E	F	G
1	**CAS**	**AMDIS Annotation**	**RT (s)**	**Environment**	**Kovats retention index**	**Golm predicted functional groups**	**BinBase splashID**
387	130405106	pentakis-O-(trimethylsilyl)-, o-methyloxyme, (1E)-	1109	Cell culture			
388	1009934	Hexamethylcyclotrisilazane	1110	Cell culture			
389	EPA-347219	>Silane, dimethyl(2-naphthoxy)dodecyloxy-	1123	Cell culture			
390	4346138	tetradecanoate	1125	Cell culture			
391	1009934	Hexamethylcyclotrisilazane	1127	Cell culture			
392	55429077	>L-Lysine, N2,N6,N6-tris(trimethylsilyl)-, trimethylsilyl ester	1128	Cell culture			
393	23743262	ethanediylbis[dicyclohexyl-	1129	Cell culture			
394	1009934	Hexamethylcyclotrisilazane	1130	Cell culture			
395	EPA-333696	trimethylsilyl-, trimethylsilyl ester	1133	Cell culture			
396	14199805	glucitol	1134	Cell culture			
397	51220736	bis(trimethylsilyl)-, trimethylsilyl ester	1139	Cell culture			
398	55255442	acid, 1-(trimethylsilyl)-, trimethylsilyl ester	1147	Cell culture			
399	55320111	(trimethylsilyl)-, trimethylsilyl ester	1151	Cell culture			

	A	B	C	D	E	F	G
1	**CAS**	**AMDIS Annotation**	**RT (s)**	**Environment**	**Kovats retention index**	**Golm predicted functional groups**	**BinBase splashID**
400	7364478	(trimethylsilyl)-, trimethylsilyl ester	1157	Cell culture			
401	55255442	acid, 1-(trimethylsilyl)-, trimethylsilyl ester	1160	Cell culture			
402	EPA-333692	trimethylsilyl-, trimethylsilyl ester	1163	Cell culture			
403	55320111	(trimethylsilyl)-, trimethylsilyl ester	1168	Cell culture			
404	EPA-192601	3,4-dicarboxylic acid, dimethyl ester	1173	Cell culture			
405	EPA-283115	>1-(1-Adamantyl)-2-tripropylsilyloxyethane	1177	Cell culture			
406	74036956	>2-Bromotetradecane	1179	Cell culture			
407	19127152	2,3,4,5-tetrakis-O-(trimethylsilyl)-, L-	1181	Cell culture			
408	18551036	(trimethylsilyl)-2,6-bis[(trimethylsilyl)oxy]-	1184	Cell culture			
409	EPA-333692	trimethylsilyl-, trimethylsilyl ester	1189	Cell culture			
410	10444072	methoxy-3-methylbenzofuran)	1202	Cell culture			
411	55520893	>Hexadecanoic acid, trimethylsilyl ester	1203	Cell culture			
412	4455269	methyl-N-octyl-	1204	Cell culture			
413	502976	>1,4-Dioxane-2,5-dione	1209	Cell culture			
414	EPA-320739	propoxycarbonyl-, tetradecyl ester	1214	Cell culture			

	A	B	C	D	E	F	G
1	**CAS**	**AMDIS Annotation**	**RT (s)**	**Environment**	**Kovats retention index**	**Golm predicted functional groups**	**BinBase splashID**
415	2582798	1,2,3,4,5,6-hexakis-O-(trimethylsilyl)-	1224	Cell culture			
416	55255442	acid, 1-(trimethylsilyl)-, trimethylsilyl ester	1225	Cell culture			
417	593453	>Octadecane	1230	Cell culture			
418	EPA-328944	methoxycarbonyl-, heptadecyl ester	1238	Cell culture			
419	55255442	acid, 1-(trimethylsilyl)-, trimethylsilyl ester	1241	Cell culture			
420	502976	>1,4-Dioxane-2,5-dione	1245	Cell culture			
421	593497	>Heptacosane	1248	Cell culture			
422	55320111	(trimethylsilyl)-, trimethylsilyl ester	1254	Cell culture			
423	17962899	(trimethylsilyl)-6-[(trimethylsilyl)oxy]-	1256	Cell culture			
424	EPA-328838	ethoxycarbonyl-, isohexyl ester	1262	Cell culture			
425	55255442	acid, 1-(trimethylsilyl)-, trimethylsilyl ester	1266	Cell culture			
426	55255442	acid, 1-(trimethylsilyl)-, trimethylsilyl ester	1267	Cell culture			
427	638368	tetramethyl-	1271	Cell culture			
428	147398445	>Undecan, 1,11-bis(9,10-dihydroanthracen-9-yl)-	1272	Cell culture			
429	EPA-333268	trimethylsilyl-, trimethylsilyl ester	1285	Cell culture			

	A	B	C	D	E	F	G
1	**CAS**	**AMDIS Annotation**	**RT (s)**	**Environment**	**Kovats retention index**	**Golm predicted functional groups**	**BinBase splashID**
430	55320111	(trimethylsilyl)-, trimethylsilyl ester	1287	Cell culture			
431	7364467	(trimethylsilyl)-, trimethylsilyl ester	1287	Cell culture			
432	EPA-307912	dimethylisopropylsilyloxy benzene	1293	Cell culture			
433	55429282	>L-Tryptophan, N,1-bis(trimethylsilyl)-, trimethylsilyl ester	1295	Cell culture			
434	EPA-379991	>Amphetamine TBDMS Derivative	1298	Cell culture			
435	EPA-328838	ethoxycarbonyl-, isohexyl ester	1306	Cell culture			
436	18748919	>Octadecanoic acid, trimethylsilyl ester	1311	Cell culture			
437	EPA-328974	Aminocyclopentanecarboxylic acid, N-	1315	Cell culture			
438	33342675	>Thiazole, 2-(phenylthio)-	1316	Cell culture			
439	55320111	(trimethylsilyl)-, trimethylsilyl ester	1317	Cell culture			
440	646060	>1,3-Dioxolane	1318	Cell culture			
441	EPA-329585	ethoxycarbonyl-, decyl ester	1325	Cell culture			
442	EPA-163596	dimethyl-N-(2'-t-butylcarbonylphenyl)-	1337	Cell culture			
443	19127152	2,3,4,5-tetrakis-O-(trimethylsilyl)-, L-	1339	Cell culture			

	A	B	C	D	E	F	G
1	**CAS**	**AMDIS Annotation**	**RT (s)**	**Environment**	**Kovats retention index**	**Golm predicted functional groups**	**BinBase splashID**
444	EPA-320830	propoxycarbonyl-, pentadecyl ester	1348	Cell culture			
445	EPA-347187	phenylprop-2-yl)phenoxy)propoxy-	1354	Cell culture			
446	21170627	diacetyl-1,4-dihydro-4-isopropyl-	1356	Cell culture			
447	593497	>Heptacosane	1359	Cell culture			
448	7364478	(trimethylsilyl)-, trimethylsilyl ester	1361	Cell culture			
449	55320111	(trimethylsilyl)-, trimethylsilyl ester	1361	Cell culture			
450	55320111	(trimethylsilyl)-, trimethylsilyl ester	1367	Cell culture			
451	55320111	(trimethylsilyl)-, trimethylsilyl ester	1368	Cell culture			
452	593497	>Heptacosane	1379	Cell culture			
453	20836413	trimethyl-N,N-bis[2-[(trimethylsilyl)oxy]ethyl	1381	Cell culture			
454	55255442	acid, 1-(trimethylsilyl)-, trimethylsilyl ester	1384	Cell culture			
455	103231	>Hexanedioic acid, bis(2-ethylhexyl) ester	1387	Cell culture			
456	EPA-315373	>Phthalic acid, di(3-methylphenyl) ester	1390	Cell culture			
457	55606741	bis[(trimethylsilyl)oxy]phenyl]ethyl]-1,1,1-trimethyl-N-	1391	Cell culture			

	A	B	C	D	E	F	G
1	**CAS**	**AMDIS Annotation**	**RT (s)**	**Environment**	**Kovats retention index**	**Golm predicted functional groups**	**BinBase splashID**
458	EPA-192601	3,4-dicarboxylic acid, dimethyl ester	1400	Cell culture			
459	EPA-329436	propoxycarbonyl-, nonyl ester	1407	Cell culture			
460	EPA-367444	>Uridine, tetra(trimethylsilyl)-	1410	Cell culture			
461	EPA-333703	trimethylsilyl-, trimethylsilyl ester	1416	Cell culture			
462	55606741	bis[(trimethylsilyl)oxy]phenyl]ethyl]-1,1,1-trimethyl-N-	1416	Cell culture			
463	EPA-328904	Aminocyclopentanecarboxylic acid, N-methoxycarbonyl-, octyl	1419	Cell culture			
464	EPA-261109	Azatricyclo[5.2.1.0(2,6)]decane-3,5-dione, 4-[4-(2-methylphenoxy)phenyl]-	1420	Cell culture			
465	14490052	>7-Methyltryptamine	1426	Cell culture			
466	35479448	disilaoctane, 2,2,7,7-tetramethyl-4,5-diphenyl-	1429	Cell culture			
467	7364445	(trimethylsilyl)-, trimethylsilyl ester	1442	Cell culture			
468	68122	dimethyl-	1443	Cell culture			
469	EPA-364699	>Ala-Gly, di (tert-butyldimethylsilyl) deriv.	1443	Cell culture			
470	EPA-192601	3,4-dicarboxylic acid, dimethyl ester	1451	Cell culture			

	A	B	C	D	E	F	G
1	**CAS**	**AMDIS Annotation**	**RT (s)**	**Environment**	**Kovats retention index**	**Golm predicted functional groups**	**BinBase splashID**
471	55606741	bis[(trimethylsilyl)oxy]phenyl]ethyl]-1,1,1-trimethyl-N-	1454	Cell culture			
472	EPA-310350	>Ethylamine, N,N-didecyl-2-(2-thiophenyl)-	1458	Cell culture			
473	55320111	(trimethylsilyl)-, trimethylsilyl ester	1459	Cell culture			
474	68595846	triTMS	1461	Cell culture			
475	55606741	bis[(trimethylsilyl)oxy]phenyl]ethyl]-1,1,1-trimethyl-N-	1467	Cell culture			
476	EPA-332747	tetrakis(trimethylsilyl) ether	1469	Cell culture			
477	EPA-363542	diethyldodecyloxy(2-methoxyethoxy)-	1477	Cell culture			
478	EPA-380438	octakis(trimethylsilyl) ether	1485	Cell culture			
479	1560970	>Dodecane, 2-methyl-	1487	Cell culture			
480	EPA-328548	(2-methoxyethoxycarbonyl)-, tetradecyl ester	1492	Cell culture			
481	EPA-380091	octakis(trimethylsilyl) ether	1496	Cell culture			
482	53294330	tetrakis(trimethylsilyl)-	1499	Cell culture			

	A	B	C	D	E	F	G
1	**CAS**	**AMDIS Annotation**	**RT (s)**	**Environment**	**Kovats retention index**	**Golm predicted functional groups**	**BinBase splashID**
483	55529685	[illegible] [2,3,4,6-tetrakis-O-(trimethylsilyl)-·-D-galactopyranosyl]-1,2,3,6-tetrakis-O-	1504	Cell culture			
484	332148911	fluorophenylethynyl)-2,3,5,6-	1509	Cell culture			
485	55429282	>L-Tryptophan, N,1-bis(trimethylsilyl)-, trimethylsilyl ester	1513	Cell culture			
486	EPA-380102	octakis(trimethylsilyl) ether, methyloxime	1520	Cell culture			
487	55255442	acid, 1-(trimethylsilyl)-, trimethylsilyl ester	1524	Cell culture			
488	EPA-322000	allyloxycarbonyl-, hexadecyl ester	1531	Cell culture			
489	EPA-380438	octakis(trimethylsilyl) ether	1541	Cell culture			
490	EPA-380438	octakis(trimethylsilyl) ether	1544	Cell culture			
491	EPA-352398	Tris(hydroxymethyl)propane, tris(trimethylsilyl)	1547	Cell culture			
492	188943	>Diindeno[1,2,3-cd:1',2',3'-lm]perylene	1554	Cell culture			
493	EPA-367439	>Guanosine, penta(trimethylsilyl)-	1557	Cell culture			
494	EPA-363093	diethylnonyloxytridecyloxy-	1563	Cell culture			

	A	B	C	D	E	F	G
1	**CAS**	**AMDIS Annotation**	**RT (s)**	**Environment**	**Kovats retention index**	**Golm predicted functional groups**	**BinBase splashID**
495	55529685	[illegible] [2,3,4,6-tetrakis-O-(trimethylsilyl)-·-D-galactopyranosyl]-1,2,3,6-tetrakis-O-	1571	Cell culture			
496	646311	>Tetracosane	1576	Cell culture			
497	EPA-380438	octakis(trimethylsilyl) ether	1577	Cell culture			
498	31123792	>1-Propanol, 2-methyl-3-(tributylstannyl)-	1580	Cell culture			
499	EPA-331943	1,2,4-triazine-3,5(2H,4H)-dione tetratms	1587	Cell culture			
500	75763	>Silane, tetramethyl-	1594	Cell culture			
501	481210	>Cholestane	1610	Cell culture			
502	EPA-281138	>d-Norandrostane (5‡,14‡)	1611	Cell culture			
503	EPA-374744	>Niacinamide, N-tert.-butyldimethylsilyl-	1615	Cell culture			
504	EPA-309180	>Sulfurous acid, butyl tridecyl ester	1616	Cell culture			
505	609234	>Phenol, 2,4,6-triiodo-	1632	Cell culture			
506	6736998	pentakis-O-(trimethylsilyl)-, D-	1645	Cell culture			
507	19127152	2,3,4,5-tetrakis-O-(trimethylsilyl)-, L-	1650	Cell culture			
508	EPA-367670	ethoxyethoxy)hexadecyloxy-	1654	Cell culture			
509	1888579	dimethyl-	1657	Cell culture			

	A	B	C	D	E	F	G
1	**CAS**	**AMDIS Annotation**	**RT (s)**	**Environment**	**Kovats retention index**	**Golm predicted functional groups**	**BinBase splashID**
510	EPA-309193	>Sulfurous acid, decyl 2-ethylhexyl ester	1695	Cell culture			
511	20633038	trimethyl-	1733	Cell culture			
512	71239151	tetrahydrofurylmethyl ester	1737	Cell culture			
513	EPA-363960	diethylheptyloxyoctadecyloxy-	1816	Cell culture			
