# Supplementary material for: Marine Metabolomics: a Method for Nontargeted Measurement of Metabolites in Seawater by Gas Chromatography–Mass Spectrometry
Source: mSystems. 2019 Dec 10;4(6):e00638-19. doi: 10.1128/mSystems.00638-19 (PMC6906741; doi:10.1128/mSystems.00638-19)
Supplement: TEXT S1 [file mSystems.00638-19-s0001.pdf]

# Supplementary Text 1. R script for peak picking for GC-MS data

```r
# Peak Picking.R
# EM Sogin
# Description: R script to pick peaks from GC-MS data

library(xcms)
library(CAMERA)

## PEAK PICKING, RETENTION TIME GROUPING & CORRECTION WITH XCMS
setwd('home/path/to/files')
files<-list.files(pattern='.mzXML', recursive = T, full.names=T)
xs <- xcmsSet(files, method = "matchedFilter", fwhm = 8.4, snthresh = 1,step= 0.25, steps= 2,sigma = 3.56718192627824, max= 500, mzdiff= 1,index= FALSE)
xset1 <- group(xs,method  = "density", bw=2, mzwid= 1, minfrac = 0.3,  minsamp = 1,max = 500) ## Initial peak grouping
xset2 <- retcor(xset1)
xset2 <- group(xset2,method  = "density", bw=2, mzwid= 1, minfrac = 0.3,  minsamp = 1,max = 500)
xset<-fillPeaks(xset2)

## Group peaks in to pseudo-spectra using CAMERA
an<-xsAnnotate(xset)
xsF<-groupFWHM(an, perfwhm=3)

peaks<-getPeaklist(xsF)
peaks[is.na(peaks)]<-0

save.image('Peak_Picking_Results.RData')
# End
```
